# Artificial intelligence-based assessment of PD-L1 expression in diffuse large B cell lymphoma

**DOI:** 10.1038/s41698-024-00577-y

**Published:** 2024-03-27

**Authors:** Fang Yan, Qian Da, Hongmei Yi, Shijie Deng, Lifeng Zhu, Mu Zhou, Yingting Liu, Ming Feng, Jing Wang, Xuan Wang, Yuxiu Zhang, Wenjing Zhang, Xiaofan Zhang, Jingsheng Lin, Shaoting Zhang, Chaofu Wang

**Affiliations:** 1 Shanghai Artificial Intelligence Laboratory, Shanghai, China; 2 Ruijin Hospital, Shanghai Jiao Tong University School of Medicine, Shanghai, China; 3 Department of Computer Science, Rutgers University, New Brunswick, NJ USA; 4 College of Electronic and Information Engineering, Tongji University, Shanghai, China; 5 Centre for Perceptual and Interactive Intelligence (CPII) Ltd. under InnoHK, HongKong, China; 6 SenseTime Research, Shanghai, China

**Keywords:** Cancer, Mathematics and computing, Lymphoma, Immunohistochemistry

## Abstract

Diffuse large B cell lymphoma (DLBCL) is an aggressive blood cancer known for its rapid progression and high incidence. The growing use of immunohistochemistry (IHC) has significantly contributed to the detailed cell characterization, thereby playing a crucial role in guiding treatment strategies for DLBCL. In this study, we developed an AI-based image analysis approach for assessing PD-L1 expression in DLBCL patients. PD-L1 expression represents as a major biomarker for screening patients who can benefit from targeted immunotherapy interventions. In particular, we performed large-scale cell annotations in IHC slides, encompassing over 5101 tissue regions and 146,439 live cells. Extensive experiments in primary and validation cohorts demonstrated the defined quantitative rule helped overcome the difficulty of identifying specific cell types. In assessing data obtained from fine needle biopsies, experiments revealed that there was a higher level of agreement in the quantitative results between Artificial Intelligence (AI) algorithms and pathologists, as well as among pathologists themselves, in comparison to the data obtained from surgical specimens. We highlight that the AI-enabled analytics enhance the objectivity and interpretability of PD-L1 quantification to improve the targeted immunotherapy development in DLBCL patients.

## Introduction

Diffuse large B cell lymphoma (DLBCL) is an aggressive blood cancer characterized by rapid progression and high incidence^1^. To better capture its tumor environment, immunohistochemistry (IHC)^2–4^ has been widely used to visualize the distribution of disease-specific proteins (e.g., PD-L1, Ki67, and CD3). In particular, assessing the programmed death ligand-1 (PD-L1) expression from IHC images is increasingly recognized as a predictive biomarker^5–8^. The significance of PD-L1 expression in solid cancers is well-documented for guiding the immune therapy, however, its impact in lymphoma is not fully explored. Emerging evidence suggests that PD-L1 checkpoint inhibitors have shown promising performance in treating lymphoma^9^. Therefore, a systematic evaluation of PD-L1 expression in DLBCL is crucial for measuring cell characterization^10^, targeted treatment^11–13^ and patient prognosis^14,15^.

The tumor proportion score (TPS)^16^ calculates the proportion of tumor cells in IHC, which is a key quantitative indicator reflecting PD-L1 expressions. Through the use of TPS, manual counting PD-L1 positive tumor cells can be time-consuming with the inter-reader variability from pathologists. Furthermore, the heterogeneous expression of PD-L1 and intrinsic characteristics of lymphoma pose significant challenges in identifying cell types and their correlations (Supplementary Section 1.1). For instance, tumor B-cells and non-malignant immune cells can co-express PD-L1 in the microenvironment. As a result, cells under positive regions (i.e., PD-L1+) may not always belong to tumor cells^17,18^. In contrast to solid tumors such as lung cancer^19–24^ and breast cancer^25,26^, where tumor regions can be deferentially identified to aid in the detection of tumor cells, PD-L1 stained whole slide images (WSIs) of DLBCL do not exhibit detectable differences between notable tumor cells and normal cells.

Growing research efforts have been made to quantify PD-L1 expression in pathology^27,28^, with its capacity to characterize non-small cell lung cancer^19–24,29^, breast cancer^25,26^, as well as head and neck squamous cell carcinoma^30^. These studies are primarily focused on well-defined tumor regions and cell traits, leading to ease the process of tumor segmentation and cell detection. These related works can be grouped into the joint analysis of HE and IHC^6,24,31^, tumor cells (TC) or immune cells (IC) scores calculation based on color threshold^10,21,25,26^, and counting tumor cells^19,22,23,29,30,32,33^.

**Fig. 1:**
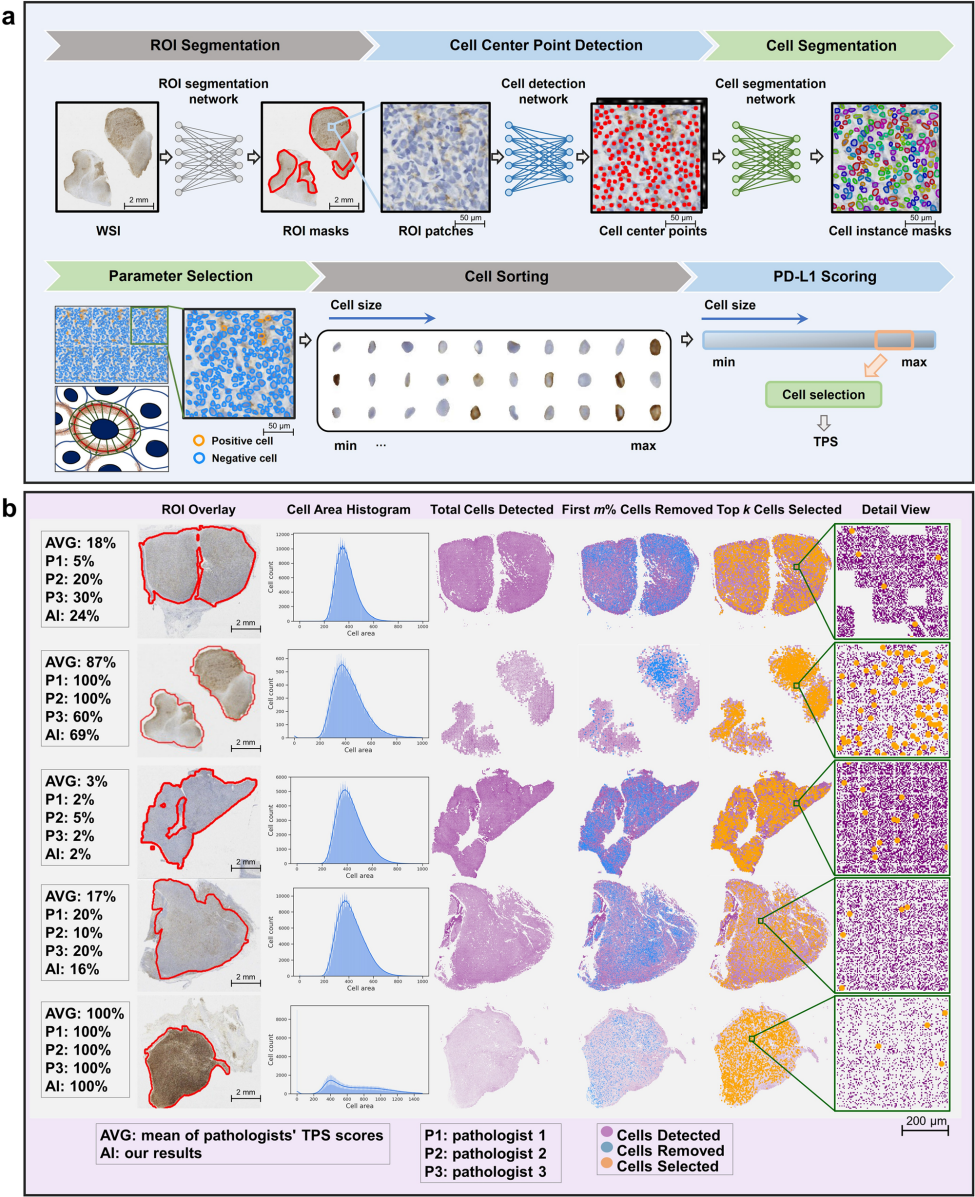
Overview and demonstration of the proposed digital PD-L1 scoring approach. **a** The pipeline includes modules for ROI segmentation, cell detection and segmentation, parameter selection, cell sorting, and PD-L1 scoring. **b** Demonstration of quantitative results and visualization depicting the distribution of cells using the proposed immunohistochemical quantitative rule.

In this study, we proposed an AI-based PD-L1 expression assessment for DLBCL patients using IHC slides (Fig. 1). To address aforementioned challenges, we made multifaceted contributions. First, we developed a PD-L1 expression scoring framework for lymphoma that accurately quantified the widely-known TPS. Second, we proposed a PD-L1 digital quantification rule, addressing the daunting challenge of digitally screening tumor cells in IHC slides for DLBCL. Finally, we constructed primary and validation cohorts with large-scale available annotations, encompassing over 5101 tissue regions and 146,439 live cells. Our study demonstrates its usefulness in quantifying the complex PD-L1 expressions, highlighting its potential to facilitate critical immunotherapies for DLBCL patients.

## Results

### Data cohorts and annotations

We collected 220 patients with DLBCL diagnosed or treated in Shanghai Ruijin Hospital from June 2019 to June 2020. Three hematopathologists diagnosed all specimens as DLBCL, germinal center B-cell subtype (GCB), or activated B-cell subtype (ABC) according to the 2016 World Health Organization Classification of Tumors in Hematopoietic and Lymphoid Tissues^34^.

**Fig. 2:**
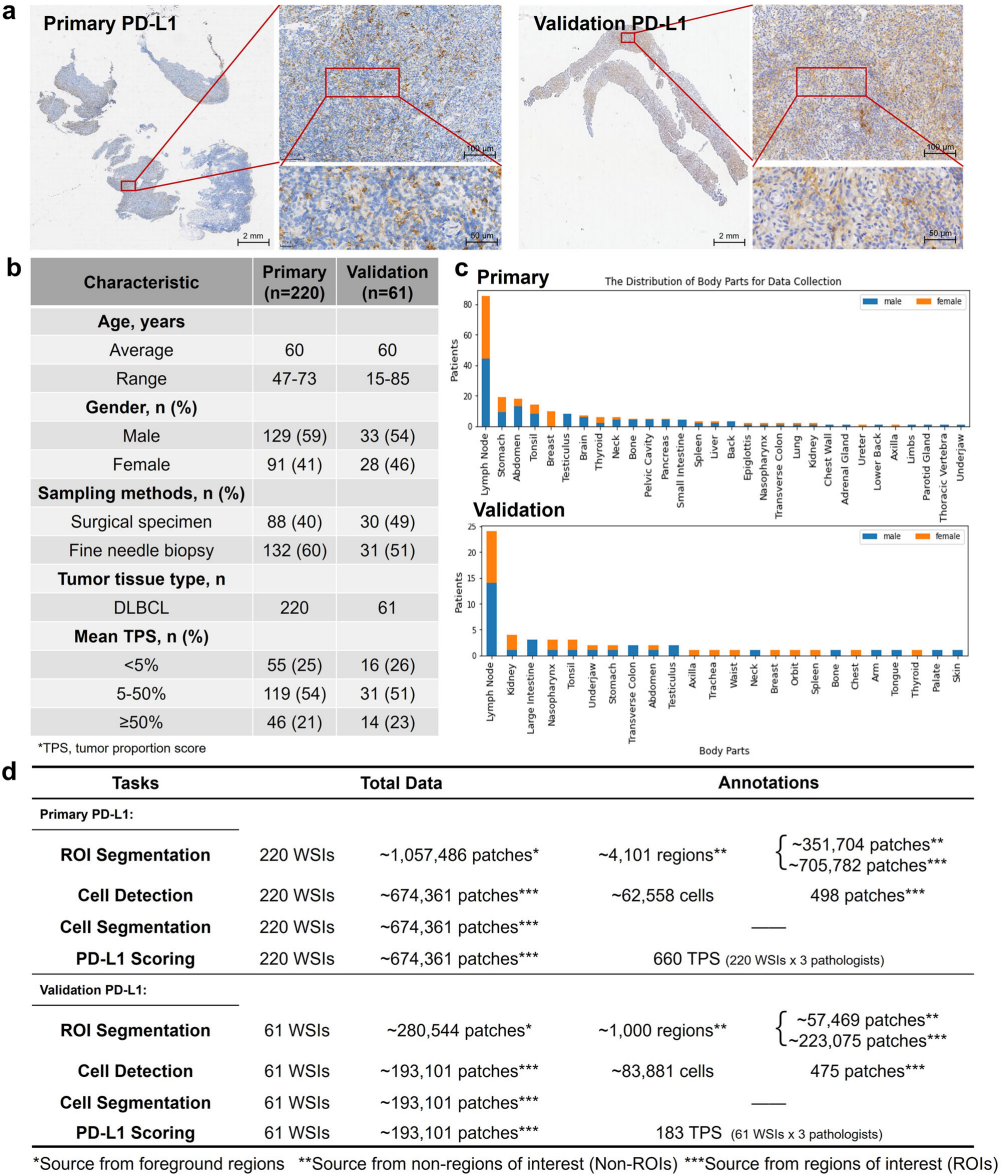
Characterization of PD-L1 Cohorts. **a** Examples of PD-L1 slide visualizations at multiple magnifications. **b** Dataset characteristics, including the mean TPS annotated by three senior pathologists under cutoffs. **c** The distribution of body parts for data collection. **d** The statistical information of PD-L1 data and annotations.

Sample staining was performed in the Pathology Department of Shanghai Ruijin Hospital. All 220 WSIs underwent immunohistochemical staining of PD-L1 (22C3, DAKO), with quality control and evaluation conducted by pathologists. The WSIs were acquired and digitized on the SQS-600P digital slide scanner at 40 × magnification. The study resulted in 220 PD-L1 stained WSIs from 30 parts of the human body, including 88 surgical specimens and 132 fine needle biopsies (Fig. 2 and Supplementary Section 1.3).

Three senior clinical pathologists (Q.D, S.J.D and Y.T.L with 15, 5, and 3 years’ experiences) scored TPS in 220 PD-L1 stained WSIs in a double-blind condition to allow the correlation measurement between pathologists and the proposed AI algorithm and the stability among pathologists. Next, a medically relevant labeling expert with 7 years’ experiences in pathology annotated the non-regions of interest (Non-ROIs) which mean non-relevant regions (Supplementary Section 1.2) for PD-L1 quantification in 220 PD-L1 stained WSIs using ASAP software^35^. These annotations resulted in a total of 4101 tissue regions from the primary cohort, encompassing areas with extrusion, burn, carbon foam, inflammation, fat, blood cells, interstitial cells, necrotic cells or debris, and other non-tumor cells. The reason for conducting this annotation step is that quantifying PD-L1 expression within regions of interest (ROIs) which mean effective regions for PD-L1 quantification is more efficient compared to analyzing the entire WSI. This is because by excluding Non-ROIs, we can reduce interference during the cell detection stage and eliminate the interference of unrelated cells in non-relevant areas. But accurately defining ROIs in PD-L1 slides for DLBCL is challenging, because blood cancer typically does not have tumor regions traditionally, primarily affecting fluid tissues as blood and bone marrow, and does not form distinct, fixed masses like solid tumors. In contrast, identifying Non-ROIs is relatively more straightforward for annotators. Since the quantitative analysis of PD-L1 expression is based on tumor regions, we applied an inversion operation to the annotated areas, thereby delineating ROIs for algorithms. Additionally, two individuals (F.Y and M.F) with medical training labeled cell center points for PD-L1 by LabelMe software^36^ and SenseCare^37^ for the iteration of the cell detection algorithm. We identified and extracted ROIs for PD-L1 quantitative analysis from each WSI, and then randomly cropped patches of size 256 × 256 at a magnification of 40×, ensuring that each patch contains more than 100 cells. We performed a uniform distribution of PD-L1 expression at the patch level, including negative, weakly positive, positive, and strongly positive areas. We also ensured that the number of patches selected from each patient’s WSI was equivalent, and there were no overlapping areas among patches. We finally obtained a total of 498 patches used for annotations of cell center points for PD-L1.

For the external validation, a total of 61 PD-L1 stained WSIs with each from a unique patient, were collected from the North Branch of Shanghai Ruijin Hospital. The patient statistics are consistent across datasets. The annotation settings of this validation cohort were aligned with those of the primary cohort (Fig. 2 and Supplementary Section 1.4). Ultimately, we obtained 61 TPS from each of the three pathologists, annotated 1000 tissue regions as Non-ROIs, and marked 475 patches for cell center points.

### Correlation and interpretability

We demonstrated the correlation between the AI algorithmic outcomes and pathologists’ results, along with vital results in PD-L1 scoring, including the performance of cell detection, segmentation and positive cell recognition.

#### Correlation analysis

**Fig. 3:**
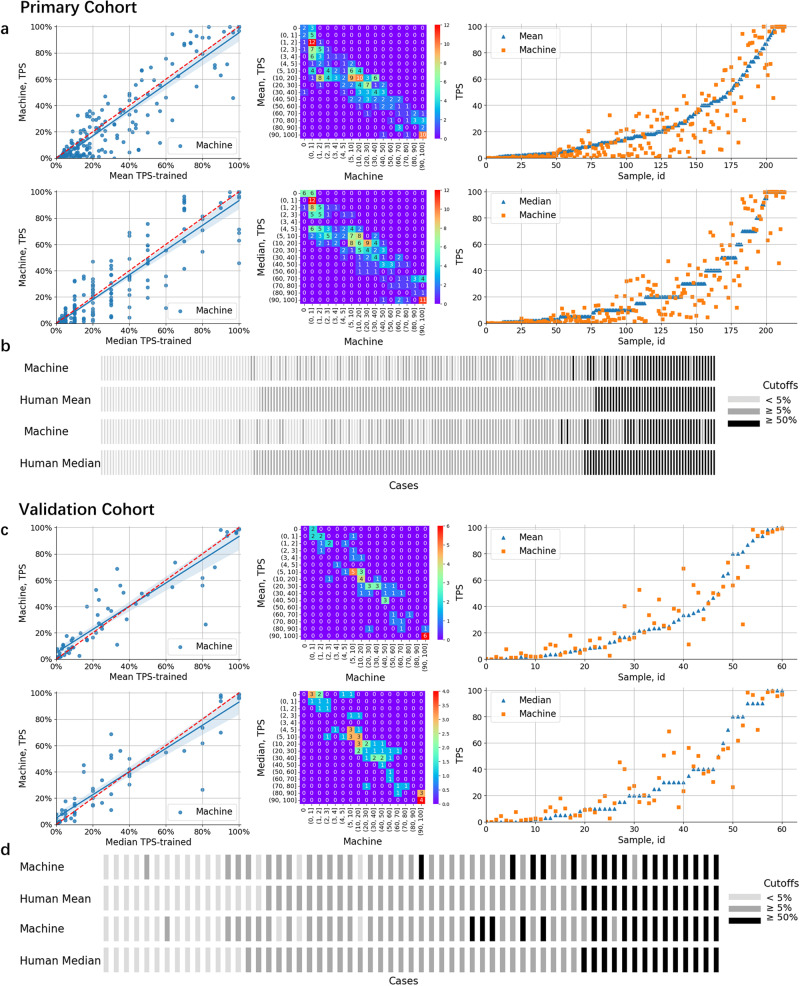
Evaluation of Model Performance in PD-L1 Quantification. **a**, **c** Correlations between the TPS values generated by the proposed algorithm and mean (or median) scores as assessed by pathologists. **b**, **d** PD-L1 expression scores were grouped into three intervals, enabling the observation of correlations between the algorithm and pathologists.

**Fig. 4:**
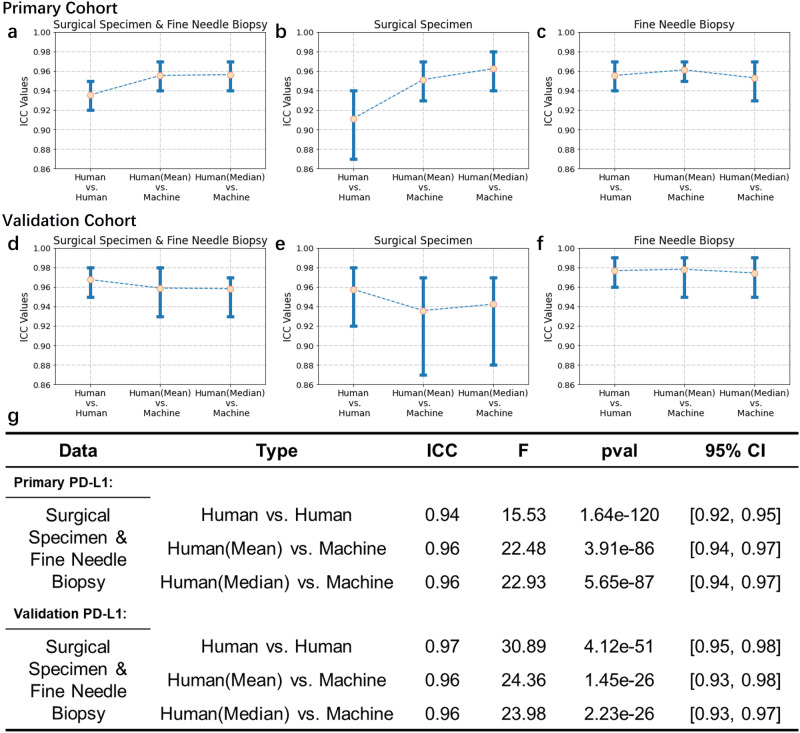
Inter-rater reliability metrics for pathological evaluations under different sampling forms. **a**–**c** Comparisons for human versus algorithm scoring in the primary cohort, across surgical specimens and fine needle biopsies. **d**–**f** The similar comparisons for the validation cohort. **g** Statistical results for PD-L1 expression between fine needle biopsies and surgical specimens.

Figures 3a, b and 4g demonstrated that the algorithm consistently provided stable quantitative findings, which were closely aligned with the mean or median scores of pathologists. Also, we observed that subjective variations in PD-L1 quantification can result in fluctuations in pathologists’ scores (Supplementary Sections 2.1.8 and 2.1.9). To mimic real-world scenarios, particularly in the relation to suggest treatment decisions, Fig. 3b categorized TPS into three stages with cutoff levels of 5% and 50% based on supported guidelines^38–40^, respectively. Furthermore, we evaluated the intraclass correlations between human raters and the algorithm using the intraclass correlation coefficient (ICC) (Fig. 4g). We recognized positive results with an intra-pathologist concordance with ICC at 0.94 (95% CI, 0.92, 0.95), while the correlation between the automated and manual scores was higher with ICC at 0.96 (95% CI, 0.94, 0.97).

#### Outcome explainability

Figure 1b presented visualization at the thumbnail level to clarify the process of digital PD-L1 quantification. Our strategy not only allowed for the precise calculation of the TPS but also provided detailed explanations. Specifically, it illustrated the area distribution of each detected cell within the ROIs, as well as the exact number of tumor cells and positive tumor cells selected for TPS calculation based on our designed PD-L1 digital quantification rules for DLBCL.

**Fig. 5:**
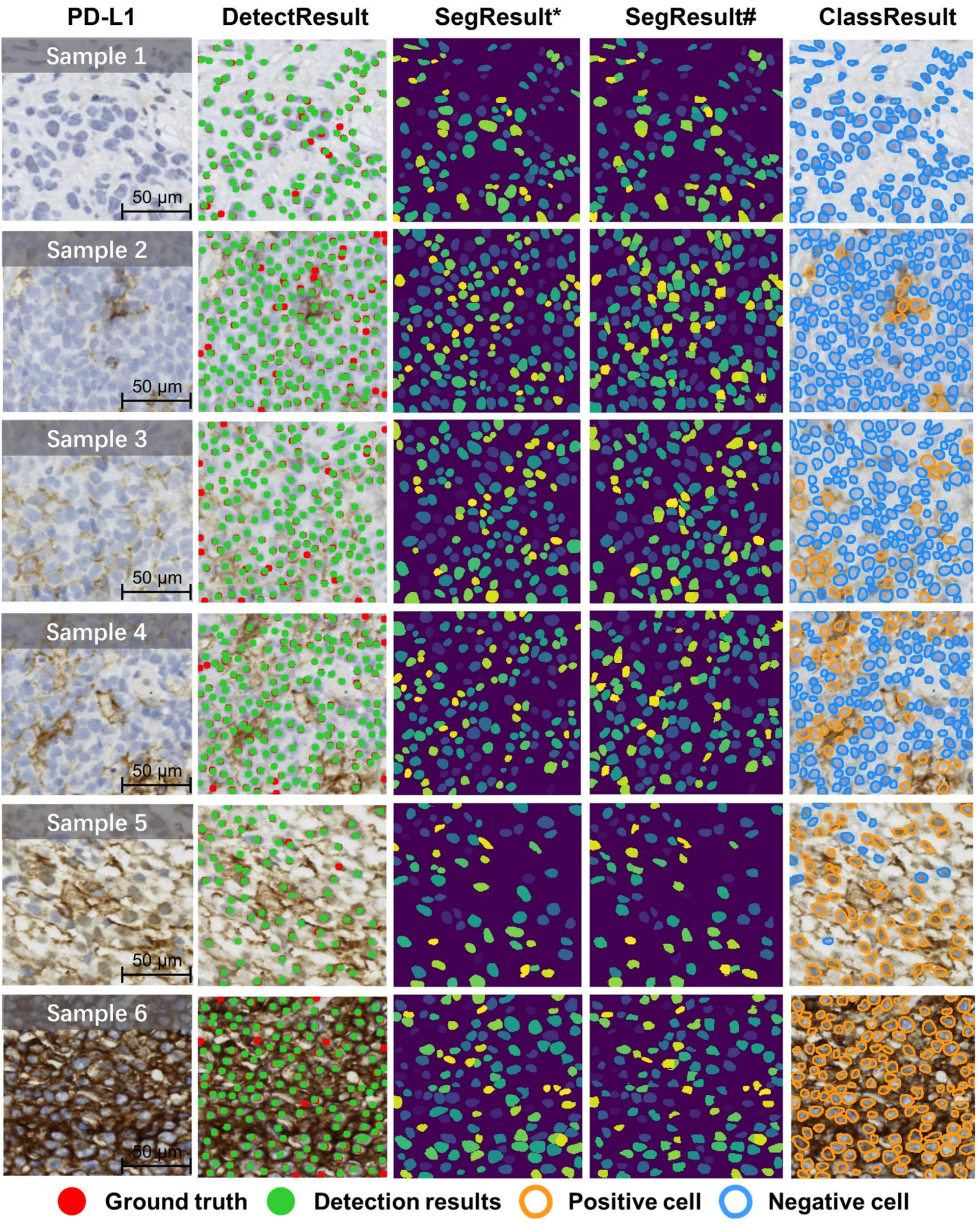
Visualizations of the cell detection, segmentation and PD-L1+ cell classification. * Two-stage segmentation result overlay, # SAM (Segment Anything) result overlay.

Figure 5 showed the performance of an integrated cell detection, segmentation and PD-L1+ cell classification framework designed for membrane-positive IHC slides. Given the impressive zero-shot performance of the image segmentation foundation model SAM (Segment Anything Model)^41^ on natural images and its vast data annotation scale of 1 billion, we also conducted experiments and discussions on the application of SAM for cell segmentation tasks in PD-L1 images (details in Supplementary Section 2.1).

**Fig. 6:**
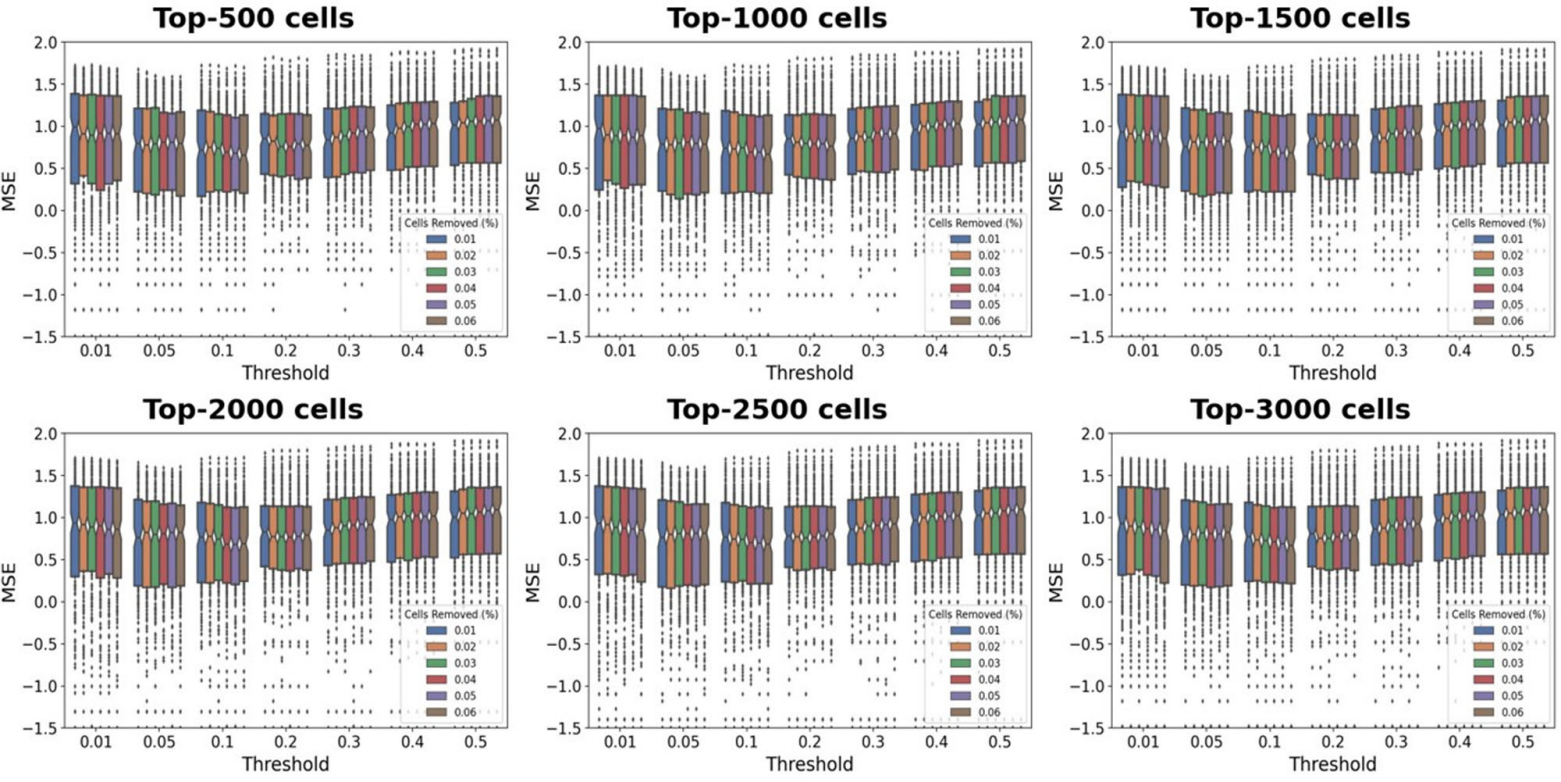
Parameter selection for proposed new PD-L1 digital quantification rules in DLBCL. Between different boxplots, each represents the impact on the MSE of selecting *k* cells. Each boxplot shows the MSE changes in logarithmic scale between the algorithm and the mean TPS assessed by pathologists under distinct thresholds (*t*%). Each bar in the boxplot at a fixed threshold represents the impact on MSE when removing the top *m*% of cells. (*k* means the selection of top cells with the largest area, *t*% means proportion of brown area in a single cell area, *m*% means the exclusion of the largest cells based on area percentage).

**Fig. 7:**
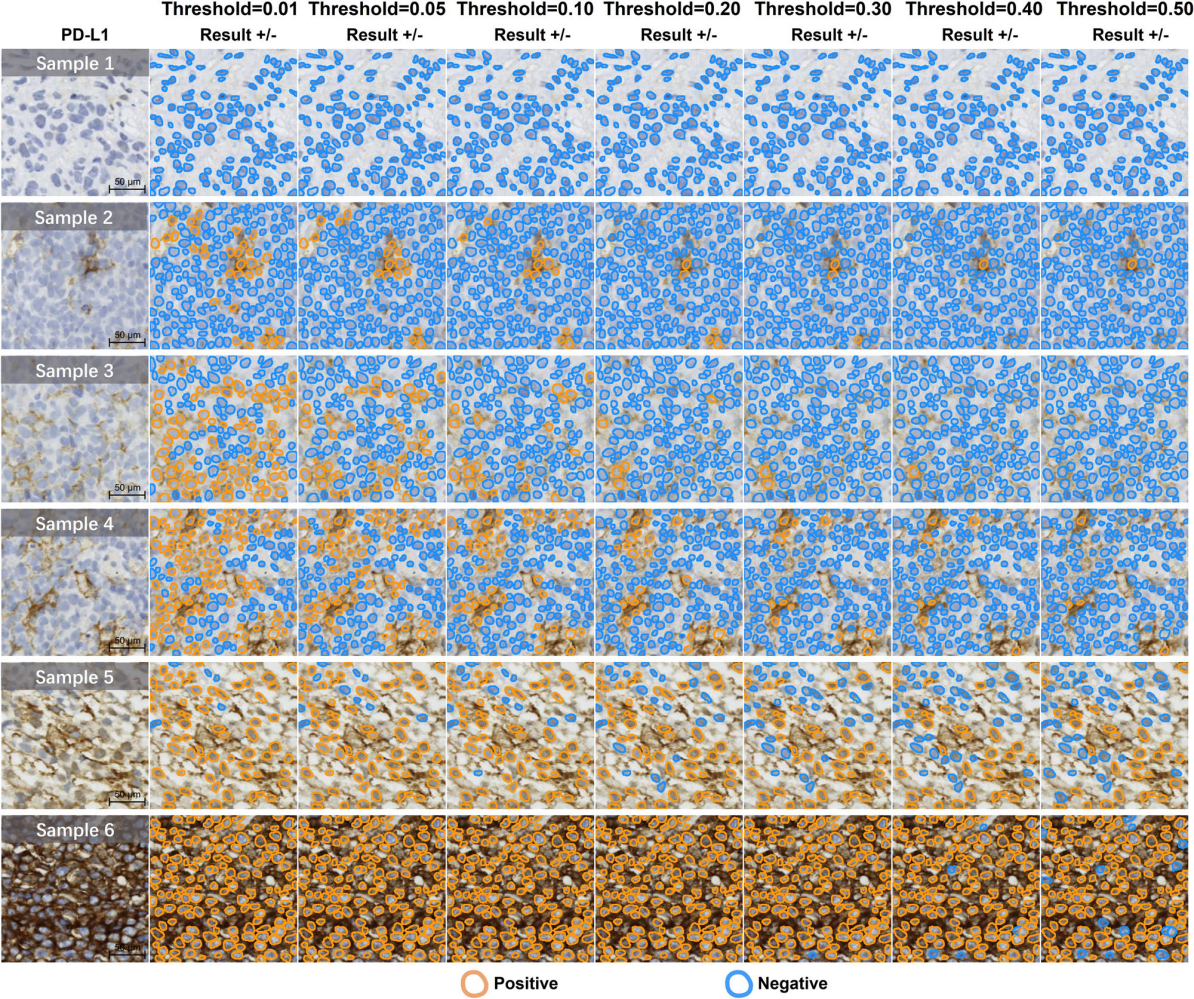
Visualizations of negative and positive cell classification across varying thresholds (*t*%). Samples 1–6 show an increasing level of PD-L1 positive expression.

Figure 6 illustrated the impact of the proposed PD-L1 digital quantification rule (Eq. (2)) for DLBCL on the recognition of positive cells in the scoring stage. We found the MSE metric exhibited a slight fluctuation trend with the variation of the parameter of cells removed *m*% (the first *m*% cells with the largest area), whereby a high *m* value affected the selection of tumor cells, whereas a low *m* value failed to adequately filter out large non-tumor cells (see the set of color bars associated with each subplot in Fig. 6, and more results in Supplementary Section 2.1.7). Figure 7 revealed that the parameter of threshold *t* (the threshold for positive or negative classification) played a key role in the proposed immunohistochemical quantitative rule, particularly on cells displaying weak or moderate PD-L1 expression (e.g., Samples 2–5 in Fig. 7). In addition, the impact of the *t* was less pronounced on cells displaying strong or no expression (e.g., Samples 1 and 6 in Fig. 7). When the *t* value was large (e.g., 0.5), the proportion of positive cells decreased, leading to a low quantitative score. Conversely, when the *t* value was small (e.g., 0.01), the proportion of positive cells increased, resulting in a high quantitative score.

### Concordance between surgical specimens and fine needle biopsies

Figures 4a–c showed the interclass ICC for PD-L1 expression between fine needle biopsies and surgical specimens. From the perspectives of both pathologists and AI models, the overall performance of PD-L1 expression scoring in fine needle biopsies is superior to that in surgical specimens (Fig. 4b–c). Specifically, for surgical specimens (Fig. 4b, the intra-pathologist concordance was found to be 0.91 (95% CI, 0.87, 0.94). The ICC between the mean scores of pathologists and the algorithm’s results was 0.95 (95% CI, 0.93, 0.97). Similarly, the ICC between the median scores of pathologists and the algorithm’s results was 0.96 (95% CI, 0.94, 0.98). In the case of fine needle biopsy (Fig. 4c), the intra-pathologist concordance was around 0.96 (95% CI, 0.94, 0.97). Moreover, the ICC between the mean scores of pathologists and the algorithm’s results was 0.96 (95% CI, 0.95, 0.97), and the ICC between the median scores of pathologists and the algorithm’s results was around 0.95 (95% CI, 0.93, 0.97), further supporting the strong agreement between pathologists and the algorithm’s assessment.

### External validation

We extend to evaluate the model performance on the external validation cohort, which presents notable variations in terms of image quality derived from scanners and DAB staining. We show the results of the algorithm’s comparison with the ratings of pathologists in PD-L1 quantification (Figs. 3c, d and 4d–f). While the algorithm’s results are slightly lower than those of pathological experts for surgical specimens (Fig. 4e), they remain relatively stable in the challenging setting with fine needle biopsies (Fig. 4f). Compared to the ratings of pathologists on TPS fluctuate, the algorithm’s results are relatively stable. In detail, the distribution is mainly around the mean or median value of pathologists’ ratings (details of cell detection and segmentation in Supplementary Section 2.2).

We further confirm that the AI algorithm exhibited higher consistency with pathologists in the evaluation of samples obtained through fine needle biopsies compared to surgical specimens. This observation is in line with the findings from the primary cohorts. Results (Fig. 4d, g) showed that the intra-pathologist concordance was 0.97 (95% CI, 0.95, 0.98). The ICC between the mean and median score of pathologists and the algorithm’s results were 0.96 (95% CI, 0.93, 0.98) and 0.96 (95% CI, 0.93, 0.97), suggesting a significant level of concordance. For surgical specimens (Fig. 4e), results showed that the intra-pathologist concordance was 0.96 (95% CI, 0.92, 0.98). The ICC between the mean and median scores of pathologists and the algorithm’s results were around 0.94 (95% CI, 0.87, 0.97) and 0.94 (95% CI, 0.88, 0.97). For fine needle biopsies (Fig. 4f), results showed that the intra-pathologist concordance was around 0.98 (95% CI, 0.96, 0.99). In addition, we have seen a strong correlation outcomes between AI and pathologists’ inputs. The ICC between the mean and median scores of pathologists and the algorithm’s results were 0.98 (95% CI, 0.95, 0.99) and around 0.97 (95% CI, 0.95, 0.99).

## Discussion

The precise assessment and evaluation of PD-L1 biomarker is crucial in the triage of cancer patients for the targeted immunotherapy. In this study, we developed an AI-enabled approach to achieve the high-level performance of characterizing PD-L1 expression in DLBCL patients. Recognizing the importance of identifying tumor cells in PD-L1 of lymphoma and counting membrane-positive cells, we proposed a new PD-L1 digital quantification rule for DLBCL to address the difficulty of tumor cell identification in expression quantification. This major discovery provides pathologists with an objective and interpretable tool for quantifying and visualizing detailed PD-L1 expression in DLBCL patients.

Our experiments demonstrated that AI-based models can provide explainable and quantitative results (Figs. 1 and 4). It is known that pathologists face limitations in accurate cell counting and calculations, especially when dealing with excessive amounts of cells. Misclassifying patients who are genuinely positive as negative could deprive them of the advantages of receiving immunotherapy. Conversely, misclassifying patients who are truly negative as positive may lead to unwarranted, costly, and potentially harmful treatments. The graded stratification (such as cutoff refers to 5% or 50%) can determine whether patients receive immunotherapy, which is in line with clinical practice and medication guidelines. Our AI-enabled analytics is able to maintain a stable and consistent performance under different cutoffs (Supplementary Sections 2.1.9 and 2.2.6).

Clinical findings^42–46^ are well documented on the task of measuring PD-L1 related surgical specimens and fine-needle biopsies, yet there is a lack of quantitative research in the field of AI. Our investigation provides deeper insights into optimizing clinical practice of specimen assessment on PD-L1 expression. Comprising fine needle biopsies and surgical specimens from two separated cohorts, our experiments identified the influence of sampling on PD-L1 quantization concordance. When examining data obtained from fine needle biopsies, there is a higher level of agreement between algorithms and pathologists, as well as among pathologists themselves, in comparison to the data obtained from surgical specimens. Importantly, the major discrepancy in our results between different samples could be attributed to the limited information provided by fine needle biopsies, whereas surgical specimens contain more noisy information (e.g., Non-ROIs) which may hinder the calculation of PD-L1 quantification.

A key contribution of our study is that the proposed framework provides an integrative pipeline involving cell detection and segmentation, resulting in highly correlated quantitative results as compared to the subjective assessment from pathologists. The two-stage cell segmentation model could streamline the process of cell sequencing and qualitative discrimination in DLBCL. In the module of cell area sequencing and quantification, our approach addresses the challenge of distinguishing membrane-positive tumor cells based on the key hypothesis that tumor cells generally exhibit larger morphologies.

Our analysis remarkably differs from prior works^6,10,21,24–26,31^ in four primary perspectives. First, unlike the joint analysis^6,31,47^ requiring the rigorous alignment of HE and PD-L1 or other IHC slides, our focus does not rely on HE slides paired with PD-L1, simplifying the workflow by reducing the high demand on whole slide preparation. Second, our analysis is well aligned with the routine diagnostic workflow for quantifying protein expression in PD-L1 images, offering a clinically-relevant and interpretable tool for pathologists. This diverges from approaches that simply relied on color threshold^10,21,25,26^ to calculate the percentage of positive area in WSI. Third, our immunohistochemical quantitative rule is specifically tailored for DLBCL as opposed to other solid tumors^19,21–26,29,30^. Finally, our experiment conducted automatic quantification on entire WSIs, rather than being limited to manually selected specific regions^22,25,29^. Using this whole-slide strategy can mimic the clinical evaluation process towards a more comprehensive and unbiased characterization of the overall PD-L1 expression.

This study has several limitations. Although showing promise of our findings, it remains necessary for prospective trial testing to validate its clinical utility for PD-L1 expression analytics with more diverse and expansive datasets. In addition, we continue to see the lack of available large-scale IHC image cell datasets. Building upon our major effort on the dataset curation, we plan to advance our investigation by constructing the high-quality, annotation-ready cell cohort derived from immunohistochemistry images. Alternatively, the use of synthetic image samples^48^ could add the potential robustness of AI models. Meanwhile, the use of foundation model evaluation could be a promising direction to improve prediction performance^49,50^. Finally, this study is limited to the development of AI models for PD-L1 expression quantification in DLBCL. New IHC quantification rules and the joint training with other immunohistochemical biomarkers, such as CD3, CD20, CD5, BCL2, BCL6, Ki67, and MUM1, would be valuable to provide more insights into diagnosis and treatment determination of lymphoma patients.

We developed an artificial intelligence-assisted framework focusing on the accurate quantification of PD-L1 expressions staining on IHC whole slide images. The digital PD-L1 quantification method we proposed significantly enhances the objectivity and interpretability of AI-based models in DLBCL patient analysis. It also ensures a high level of consistency between AI-generated insights and pathologists’ evaluations. Taken together, our findings show that pathologists of different levels of experience can potentially benefit from the AI-assisted model to enhance the immunotherapy decision making for DLBCL patients.

## Methods

We proposed an automated framework for PD-L1 expression quantification in DLBCL, along with a designed PD-L1 digital quantification rule for DLBCL to address the challenge of accurately identifying tumor cells in quantifying PD-L1 expression of lymphoma. Illustrated in Fig. 1a, the expression scoring framework comprises four major components, which includes: (1) Segmenting the regions of interest that significantly impact PD-L1 expression quantification. (2) Detecting and precisely segmenting individual cells. (3) Classifying positive and negative PD-L1 cells. (4) Utilizing the proposed PD-L1 digital quantification rule for DLBCL to facilitate automated PD-L1 scoring analysis (more details in Supplementary Section 3).

The proposed approach is founded upon a critical morphological characteristic of DLBCL cells, which is the typical presentation of B lymphocytes in DLBCL as having a medium to large size. The nuclei of these lymphocytes are either equal to or larger than the size of normal macrophages, or more than twice the size of normal lymphocytes^1^. Conversely, smaller cells may be indicative of normal lymphocytes or smaller tumor cells.

The study was conducted in accordance with the Declaration of Helsinki, and was approved by the Committees on Human Research of Shanghai Ruijin Hospital. Informed consent was obtained for each patient.

### ROI segmentation

We developed a ROI segmentation framework that treated the task as binary classification at the patch level. Each patch was generated through sliding cropping of WSIs. We partitioned each WSI into multiple patchesand the algorithm processed all cropped patches, assigning each as inside and outside of ROIs. The patches cropped from each WSI can be reassembled back into a complete WSI slide based on their spatial coordinate mappings. With each patch carrying a binary classification label, we effectively delineated ROIs at the WSI level. We fine-tuned a series of classification models using pre-trained models, including Densenet121^51^, Resnet18^52^, and Vision Transformer (ViT)^53^. Given their performance (as detailed in Supplementary Section 2.1.2), we opted for the fine-tuned ViT model as our primary choice for ROI segmentation. This model is capable of identifying ROIs in DLBCL disease within PD-L1 slides. Subsequently, our focus shifted towards cell detection and segmentation.

### Cell detection and segmentation

Our primary objective was to acquire the detailed cell instance information (e.g., location, area and shape of cells), essential for building a new PD-L1 digital quantification rule for DLBCL. However, there is a lack of publicly available datasets containing positive cell membrane immunohistochemical data, especially for the PD-L1 biomarker. Additionally, directly employing HE slides for PD-L1 slide segmentation proved impractical due to the substantial interference from positive cell membranes. To address these challenges, we employed a two-step approach. Initially, we employed automated cell detection for efficient annotations of cell center points. Subsequently, we incorporated a point-to-mask segmentation network for each cell. This network model facilitated the generation of detailed cell masks, providing crucial information regarding cell shape and area.

We used the AuxCNN^54^ model as a robust cell detection backbone based on a concatenated fully convolutional regression network (C-FCRN)^55^. AuxCNN refers to auxiliary convolutional neural networks employed to assist in the training of intermediate layers of a density regression model designed for automatic cell counting in microscopy images and HE. By utilizing manually annotated PD-L1 cell center points as the reference ground truth, we retrained a model for detecting PD-L1 cell center points, using the AuxCNN framework.

Following cell detection results obtained from AuxCNN (visible in the second column of Fig. 5 and Supplementary Section 2.1.5), we utilized these detected cell center point locations as input for the pre-trained cell segmentation model. Our primary objective was to enhance the precision of PD-L1 cell segmentation while mitigating interference from the positive cell membrane. Specifically, we employed NuClick^56^ as an interactive point-to-mask instance segmentation model. To facilitate comparative segmentation analysis and exploring foundation models, we integrated SAM^41^ with PD-L1. SAM is a foundation model^57–60^ for image segmentation adaptable to various scenarios^61,62^. It offers prompt customization such as points, boxes, masks, and text, eliminating the need for additional training across various natural visual segmentation tasks. Although neither NuClick nor SAM has been explicitly fine-tuned for PD-L1 data analysis, the HE dataset named MoNuSeg^63,64^ used in NuClick shares the hematoxylin staining channel with PD-L1. This is medically instructive for cell nuclei segmentation, leading us to ultimately choose NuClick as our cell segmentation method.

Following the segmentation of cell nuclei, we applied a dilation operation to the segmentation mask of these nuclei to identify positive and negative cells in the subsequent step (Supplementary Section 3.3). The objective was to expand the coverage area of the masks to encompass more than an individual size of membrane whenever possible. This approach offers the advantage of more accurately conforming to the actual cell shape, thereby enhancing the accuracy of positive cell identification.

### Positive and negative cell classification

The identification of positive and negative cells is a crucial step in quantifying PD-L1 expression. We here define cells with a brownish membrane after DAB staining as positive cells (PD-L1+ cells). However, due to the dense arrangement of cells in actual images, complete membrane enclosures for individual cells are often not observed. Instead, we encounter notable cases of both intact and incomplete membranes, as well as cells displaying strong positive, moderate positive, weak positive, and negative states. To enhance the accuracy of cell classification, we focus on the cell segmentation contours rather than relying on fixed-size regions (e.g., rectangles or circles) around the cell’s center point. By dilating a specific outward region, we calculated the ratio of the brown area within the dilated cellular membrane boundary to the total area within this boundary. This approach helps in better determining the positive or negative status of cells. Next, our objective is to identify PD-L1+ tumor cells among the identified positive and negative cells.

### New PD-L1 digital quantification rules for DLBCL

We proposed a new method for calculating PD-L1 digital quantification rules for DLBCL based on three factors: (1) Not all cells within the ROIs are tumor cells, there are also cells such as macrophages. (2) The difficulty in determining certain cell categories in PD-L1 slides. (3) Identifying the cell category of a specified area interval based on the nature of the large nucleus of tumor cells. Our primary goal is to identify live tumor cells and PD-L1+ tumor cells at a certain ratio through the cell screening process, for the calculation of TPS.

Specifically, we divided all cells within ROIs from a WSI and sorted them based on areas of the cell nucleus. However, as the cells with the largest areas might be non-tumor cells such as macrophages or vascular endothelial cells, we removed the top *m*% of cells with the largest areas. The remaining cells were then sorted by the number of top-*k* PD-L1+ tumor cells, which were identified as the tumor cells.

Using the strategies mentioned above, the traditional TPS was calculated using Eq. (1), and the TPS^*^ after applying the new PD-L1 digital quantification rule for DLBCL was shown in Eq. (2).

1 $$\,{{\mbox{TPS}}}=\frac{{{\mbox{Number of PD-L1 stained tumor cells}}}}{{{\mbox{Number of viable tumor cells}}}\,}\times 100 \%$$

2 $${{{\mbox{TPS}}}}^{* }={{\mbox{Number of PD-L1 stained tumor cells in top-$k$ cells}}}/{{\mbox{$k$}}}\,\times 100 \%$$

### Experimental settings

#### Evaluative criteria

For correlation analysis, ICC3k was used to reflect the means of a fixed set of *k* raters for each target. The F statistic, numerator degrees of freedom (df1), denominator degrees of freedom (df2), *p*-value (pval), and 95% confidence intervals (95% CI) were reported.

Given that 6 slides from the primary cohort exhibited insufficient cell counts for TPS calculation, a total of 214 slides were ultimately incorporated into the outcome assessment, although the whole pipeline was applied to all 220 slides. Moreover, 61 slides from the validation cohort were fully involved in all calculations.

#### Parameters in PD-L1 scoring

The three major parameters were *m*, *k*, and *t* (Eq. (1)), which corresponded to the exclusion of the first *m*% cells with the largest area, the selection of the top-*k* cells with the largest area for each WSI, and the proportion of brown area in a single cell area, represented as *t*%. These parameters determined the degree of PD-L1 expression detected in cells and the final TPS output produced by the algorithm. To determine the appropriate parameters, we compared the results of quantification and visualization with the mean scores of pathologists and then selected parameters that provided satisfactory performance. Specifically, we selected *m* of 0.06, *k* of 3000, and *t* of 0.1.

#### Training configurations

In the process of segmenting ROIs and identifying cells, we employed a five-fold, cross-validation strategy. For cell segmentation, we utilized a pretrained model for inference as there was no available ground truth data on cell annotations. The data split settings with validation, training, test, or external sets were shown in Supplementary Sections 1.3 and 1.4. We always used the same hyperparameters in all internal and external experiments. We trained the ROI segmentation model based on ViT of tiny version with a batch size of 256 and a learning rate at the beginning of 3*e*^−4^, for a total of 30 epochs. We trained the cell-point detection model based on a batch size of 256 and a learning rate at the beginning of 3*e*^−4^, for a total of 90 epochs.

#### Hardware and software

All data were stored and processed on our in-hourse computing servers with graphics processing units (GPUs), and central processing units (CPUs). There is a cluster of 96 Inter Xeon CPUs and 8 A100 GPUs scheduled by a Slurm system with NAS storage. We used Python 3.9 with Pytorch 1.8 to train and test the models. All code was developed using open-source tools and packages.

### Reporting summary

Further information on research design is available in the Nature Research Reporting Summary linked to this article.

### Supplementary information

REPORTING SUMMARY

Supplementary information

## Data Availability

The datasets used in this study are not publicly available at this moment due to data usage agreement restrictions. The de-identification process and usage of slides have been approved by Shanghai Artificial Intelligence Laboratory and Shanghai Ruijin Hospital. The datasets are available upon reasonable request and in order to use the data, qualified researchers should be approved by the institutional review boards of both institutions.

## Code availability

The code can be used only for non-commercial purpose and under the permission of the corresponding authors. Source code used in this study can be found at https://github.com/yanfang-research/Digital-PD-L1-Scoring.
