# Supplementary material for: Artificial intelligence-based assessment of PD-L1 expression in diffuse large B cell lymphoma
Source: NPJ Precis Oncol. 2024 Mar 27;8:76. doi: 10.1038/s41698-024-00577-y (PMC10973523; doi:10.1038/s41698-024-00577-y)
Supplement: Supplementary file 2 — Supplementary information [file 41698_2024_577_MOESM2_ESM.pdf]

Artificial Intelligence-Based Digital Assessment of PD-L1 Expression in Diffuse Large B Cell Lymphoma

Contents

1 Data Profile (PD-L1) **4**

1.1 Supplementary Figure 1: Double-blind labels of cell classification reveals difficulty identifying DLBCL tumor cells in PD-L1 images 4

1.2 Supplementary Figure 2: Annotation example in ROI Segmentation stage 4

1.3 Supplementary Table 1: Data statistics of primary cohort . . . 5

1.4 Supplementary Table 2: Data statistics of validation cohort . . 5

2 Result Supplement **6**

2.1 Primary Cohorts (PD-L1) 6

2.1.1 Supplementary Result 1: Detection and segmentation. . 6

2.1.2 Supplementary Table 3: ROI segmentation model performance 8

2.1.3 Supplementary Table 4: Cell detection model performance 8

2.1.4 Supplementary Figure 3: Visualizations of ROI segmentation in PD-L1 images 9

2.1.5 Supplementary Figure 4: Visualizations of cell centerpoint detection in PD-L1 images 10

2.1.6 Supplementary Figure 5: Visualizations of cell segmentation in PD-L1 images 11

2.1.7 Supplementary Figure 6: MSE between the algorithm results and the median scores of clinical pathologists for TPS in logarithmic scale. 12

2.1.8 Supplementary Figure 7: Correlations between algorithm or pathologist results and mean, median scores of clinical pathologists. 13

2.1.9 Supplementary Figure 8: Visualizations of correlations between algorithm or pathologist results and mean, median scores of clinical pathologists. 14

2.2 Validation Cohorts (PD-L1) 15
2.2.1 Supplementary Figure 9: Visualizations of ROI segmentation in PD-L1 images. 15
2.2.2 Supplementary Figure 10: Visualizations of cell centerpoint detection in PD-L1 Images. 16
2.2.3 Supplementary Figure 11: Visualizations of cell segmentation in PD-L1 images. 17
2.2.4 Supplementary Figure 12: Visualizations of negative and positive cell classification under various parameter thresholds. 18
2.2.5 Supplementary Figure 13: Correlations between algorithm or pathologist results and mean, median scores of clinical pathologists. 18
2.2.6 Supplementary Figure 14: Visualizations of correlations between algorithm or pathologist results and mean, median scores of clinical pathologists. 20
2.2.7 Supplementary Figure 15: The interclass correlation coefficients (ICC) for PD-L1 expression between fine needle biopsies and surgical specimens 20

3 Methodology Supplement **21**
3.1 Detailed Summary 21
3.2 Supplementary Figure 16: Detailed description for multi-stage PD-L1 quantization. 22
3.3 Supplementary Figure 17: Dilation example in identifying positive cells. 22

4 CD3 and CD10 **23**
4.1 Data Profile 23
4.1.1 Supplementary Table 5: Data statistics of CD3 23
4.1.2 Supplementary Table 6: Data statistics of CD10 23
4.1.3 Annotations 23
4.2 Result Supplement 24
4.2.1 Supplementary Figure 18: Visualizations of cell centerpoint detection (CD3) 24
4.2.2 Supplementary Figure 19: Visualizations of cell segmentation (CD3) 25
4.2.3 Supplementary Figure 20: Visualizations of negative and positive cell classification under different parameter thresholds (CD3) 26
4.2.4 Supplementary Figure 21: Visualizations of cell centerpoint detection (CD10) 27
4.2.5 Supplementary Figure 22: Visualizations of cell segmentation (CD10) 28

4.2.6 Supplementary Figure 23: Visualizations of negative and positive cell classification under different parameter thresholds (CD10) 29

1 Data Profile (PD-L1)

1.1 Supplementary Figure 1: Double-blind labels of cell classification reveals difficulty identifying DLBCL tumor cells in PD-L1 images

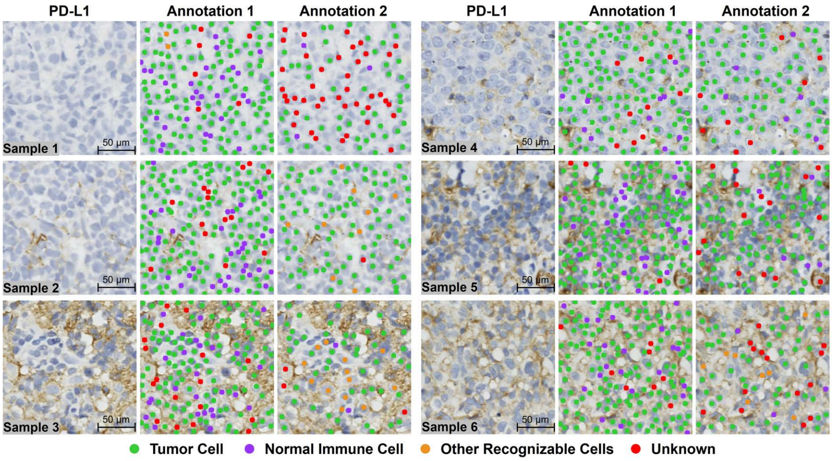

Fig. 1 Two pathologists classified PD-L1 data into cell types in a double-blind manner, dividing the cells into four categories: tumor cells, normal immune cells, other identifiable cells, and unknown cells, marked in green, purple, yellow, and red, respectively. Six examples are presented above and by comparing the results annotated by the two pathologists, it was found that identifying tumor cells of the DLBCL in PD-L1 images is highly challenging. This example demonstrates that it is difficult to devise effective algorithms using conventional PD-L1 quantification methods.

1.2 Supplementary Figure 2: Annotation example in ROI Segmentation stage

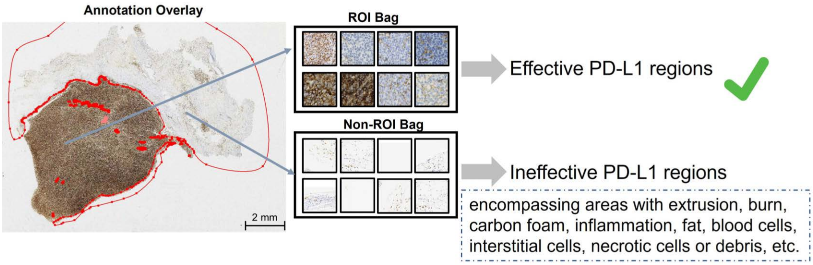

Fig. 2 Annotation example in ROI segmentation stage. Patches from the ROI bag will be utilized in the quantitative analysis of PD-L1 for algorithms.

1.3 Supplementary Table 1: Data statistics of primary cohort

Tasks	Annotations		5-fold Cross-validation			Patients
**ROI Segentation**	220 WSIs	∼4,101 regions	Train	132 WSIs	∼634,491 patches	220
			Eval	44 WSIs	∼211,497 patches	
			Test	44 WSIs	∼211,498 patches	
			Infer	220 WSIs	∼1,057,486 patches	
**Cell Detection**	498 patches	∼62,558 cells	Train	132 WSIs	∼296 patches	220
			Eval	44 WSIs	∼104 patches	
			Test	44 WSIs	∼98 patches	
			Infer	220 WSIs	∼674,361 patches	
**Cell Segmentation**	-		Infer	220 WSIs	∼674,361 patches	220
**PD-L1 Scoring**	660 TPS from 3 pathologists		220 WSIs			220

Table 1 Data Statistics of primary PD-L1 cohort.

1.4 Supplementary Table 2: Data statistics of validation cohort

Tasks	Annotations		External Validation			Patients
**ROI Segmentation**	61 WSIs	∼1,000 regions	Infer	61 WSIs	∼280,544 patches	61
**Cell Detection**	475 patches	∼83,881 cells	Infer	61 WSIs	∼193,101 patches	61
**Cell Segmentation**	-		Infer	61 WSIs	∼193,101 patches	61
**PD-L1 Scoring**	183 TPS from 3 pathologists		61 WSIs			61

Table 2 Data Statistics of validation PD-L1 cohort.

2 Result Supplement

2.1 Primary Cohorts (PD-L1)

2.1.1 Supplementary Result 1: Detection and segmentation.

ROI Segmentation. We compared the training performance of Densenet121[1], Resnet18[2], and ViT[3] models and ultimately selected the ViT model due to the performace and novelty (Table 3). Using the pretrained-finetune ViT for PD-L1 classification resulted in an ACC of 0.80 ± 0.03, AUC of 0.86 ± 0.04, F1 score of 0.84 ± 0.03, recall of 0.83 ± 0.03, precision of 0.86 ± 0.05, and Dice score of 0.79 ± 0.01. Figure 3 presented the results of ROI segmentation at the WSI level. Notably, PD-L1 images typically included control samples from other individuals or tissues for the control test of the staining effect of PD-L1 immunohistochemical. We automatically removed these control samples in both the manual annotation and pre-training network stages.

Cell Detection. The results indicated the excellent performance of the cell detection algorithm in PD-L1 images, even when dealing with densely packed and interconnected nuclei, regardless of whether the images were negative or strongly positive. However, certain factors such as the presence of positive cell membrane, lower image quality compared to HE, and unclear nuclei can significantly interfere with cell detection accuracy. Upon retraining the AuxCNN model using PD-L1 data, the resulting model achieved impressive metrics: a mean average error (MAE) of 5.66 ± 1.21, a mean average error of the density map (MACE) of 5.97 ± 1.74, a game-16 error (GAME-16) of 0.80 ± 0.05, and a game-64 error (GAME-64) of 0.30 ± 0.02, PDE (x-axis) of 0.55 ± 0.02, PDE (y-axis) of 0.56 ± 0.01), and EDA of 9.36 ± 5.49) (Table 4). We showcased more results of cell detection at the patch level (Figure 4).

Cell Segmentation. The results on demonstrated the effectiveness of the cell segmentation algorithm in PD-L1 images, especially when the interference from positive cell membrane was minimal. However, it should be noted that strong membrane positivity can significantly disrupt the accurate segmentation of the cell nucleus, as the visually observed brown cell membrane posed a challenge. Consequently, the algorithm focused on segmenting only the visible portion of the nucleus rather than the entire nucleus. To provide visual evidence, we presented the results of cell segmentation at the patch level (Figure 5) using both the NuClick and segment anything model (SAM) [4], which served as the foundational image segmentation model. In the case of the SAM model, we leveraged the preceding step of cell detection by employing a 12×12 bounding box, expanded from the center point of cell detection, as the prompt for SAM. This approach was selected to mitigate the risk of encountering complete failures that may arise from using solely center points or a combination of points and bounding boxes as prompts. It was important to highlight that due to the scarcity of publicly available PD-L1 datasets with cell segmentation annotations, acquiring ground truth (GT) data for PD-L1 cell segmentation

proved to be a challenging task. Therefore, we did not calculate evaluation indicators specifically for cell segmentation.

Evaluative Criteria. For ROI segmentation in WSI, we evaluated the performance of our two classification model using various metrics, including accuracy (ACC), precision, recall, F1-score, area under the curve (AUC) at the patch level, calculated from the number of true positives (TP), false positives (FP), false negatives (FN), and true negatives (TN) as (TP+TN)/(TP+TN+FP+FN), TP/(TP+FP), TP/(TP+FN), and $(2 \times \text{Precision} \times \text{Recall})/(\text{Precision}+\text{Recall})$, respectively. Additionally, we evaluated the performance of ROI segmentation between the predicted mask (Pred) and annotated mask (GT) at the WSI level using the Dice coefficient as $2\|\text{Pred} \cap \text{GT}\|/(\|\text{Pred}\| + \|\text{GT}\|)$.

For cell detection, we assessed the performance of cell detection using several metrics, including the mean average error (MAE), mean average absolute error of cell density estimation (MACE), and the global absolute mean error within local windows of size 32×32 or 64×64 using the GAME-32 and GAME-64 metrics, respectively. Pointwise Discrepancy Evaluation (PDE) on axis-x and axis-y, and Euclidean Distance Analysis (EDA). Specifically, MAE calculated the average absolute difference between the predicted and GT cell counts across all validation images, while MACE did the same but for the estimated cell density maps. Notably, GAME-32 and GAME-64 computed the mean absolute difference between the predicted and GT cell counts within local windows of the specified sizes for all validation images. PDE compared GT and predicted cell centers point-by-point, calculating Manhattan distance for axis-x and axis-y coordinates to identify dimensional biases. EDA calculated and averaged Euclidean distances between each pair of GT and predicted cell centers, assessing overall 2D spatial accuracy.

2.1.2 Supplementary Table 3: ROI segmentation model performance

Data	Method	ACC	AUC	F1	Recall	Precision	Dice
Primary cohort	Densenet	0.76±0.03	0.82±0.04	0.81±0.03	0.79±0.03	0.84±0.05	0.77±0.02
Primary cohort	Resnet	0.77±0.03	0.83±0.04	0.82±0.03	0.80±0.04	0.84±0.05	0.77±0.02
Primary cohort	ViT	0.80±0.03	0.86±0.04	0.84±0.03	0.83±0.03	0.86±0.05	0.79±0.01
Validation cohort	ViT	0.74±0.00	0.76±0.00	0.83±0.00	0.77±0.00	0.90±0.00	0.76±0.00

Table 3 ROI Segmentation Model Performance.

2.1.3 Supplementary Table 4: Cell detection model performance

Data	Modality	MAE	MACE	GAME-16	GAME-64	PDE (x-axis)	PDE (y-axis)	EDA
Primary cohort	PD-L1	5.66±1.21	5.97±1.74	0.80±0.05	0.30±0.02	0.55±0.02	0.56±0.01	9.36±5.49
Validation cohort	PD-L1	3.93±0.00	5.41±0.00	0.69±0.00	0.28±0.00	0.47±0.00	0.48±0.00	4.29±0.00
-	CD3	8.09±1.11	8.41±1.81	1.09±0.08	0.44±0.03	0.91±0.12	0.93±0.12	14.79±3.74
-	CD10	8.86±1.50	9.05±2.85	1.09±0.13	0.43±0.06	0.59±0.05	0.59±0.05	9.18±4.93

Table 4 Cell Detection Model Performance.

2.1.4 Supplementary Figure 3: Visualizations of ROI segmentation in PD-L1 images

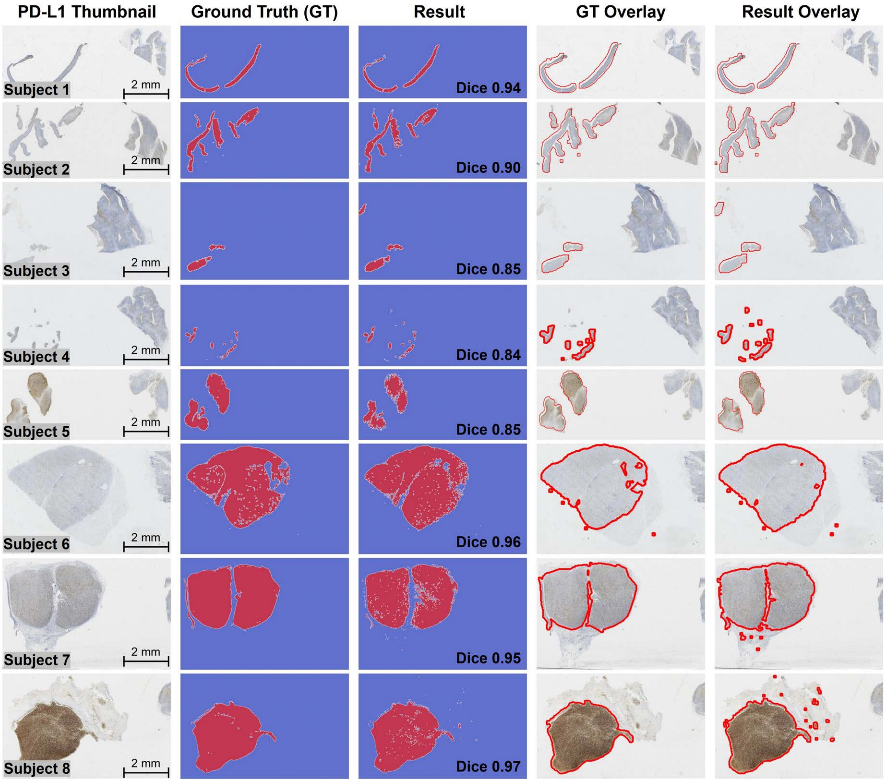

Fig. 3 Visualizations of ROI Segmentation in PD-L1 Images. This figure shows 8 sets of whole-slide image (WSI)-level results, selected from the primary dataset, in rows, with each row containing samples in the form of surgical specimens or fine needle biopsies.

2.1.5 Supplementary Figure 4: Visualizations of cell center-point detection in PD-L1 images

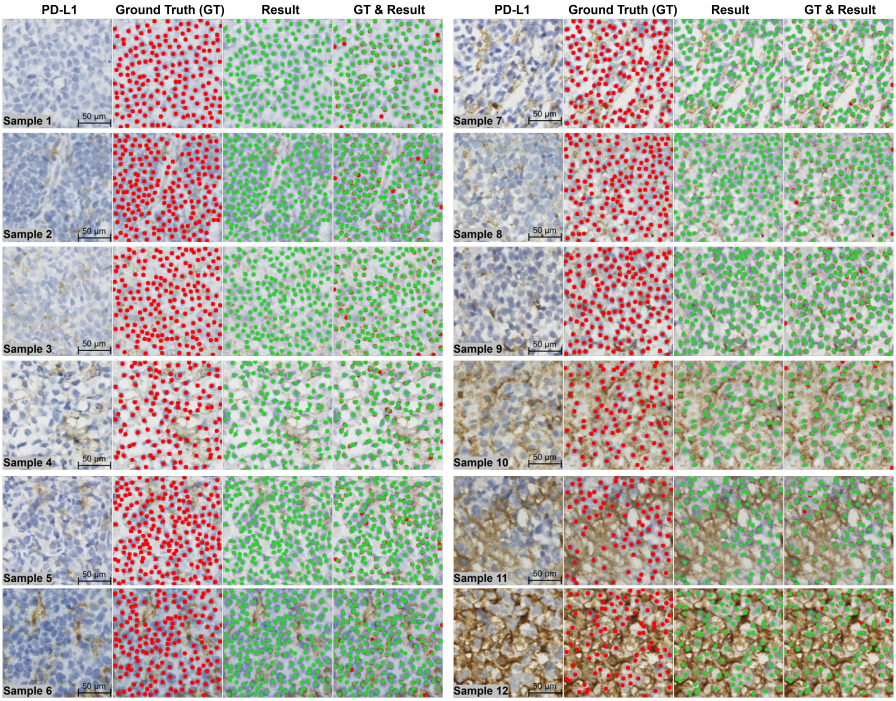

Fig. 4 Visualizations of Cell Center-point Detection in PD-L1 Images. A total of 12 groups of patch-level results, ranging from negative to strongly positive PD-L1 expression, were selected from the test set to display. Each set of results includes four columns, which are the original image, GT annotated by individuals with medical training, algorithm results, and the overlay of GT and algorithm results. Red and green dots are used to indicate the locations of cell centers detected by GT and algorithm results, respectively.

2.1.6 Supplementary Figure 5: Visualizations of cell segmentation in PD-L1 images

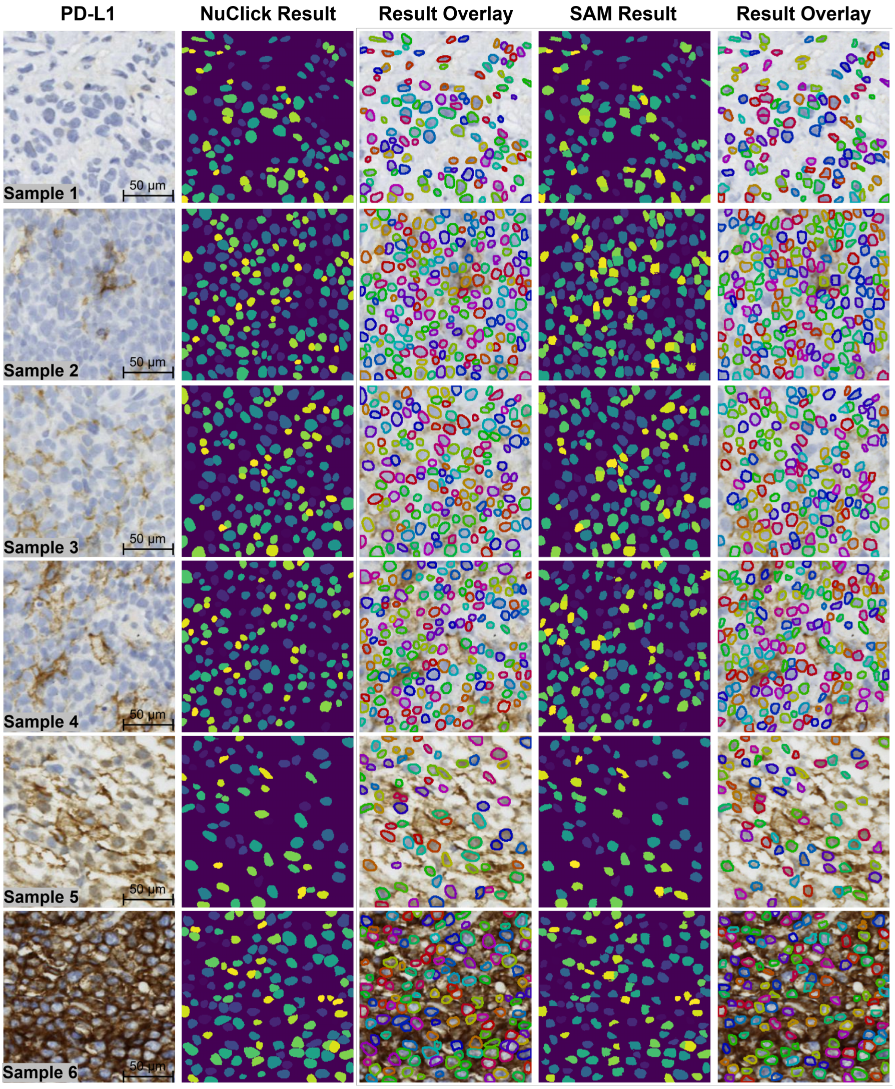

Fig. 5 Visualizations of Cell Segmentation in PD-L1 Images. This figure displays 6 groups of patch-level results, representing negative, weak positive, and strongly positive PD-L1 expression, selected from the test set. Each group of results includes three columns: the original image, the algorithm's segmentation results, and the overlay of the segmentation results and the original image from NuClick and SAM models (arrows indicate the differences between the results of two models). The different colors of the cell masks and contours are used solely for visualization purposes in the cell instance segmentation.

2.1.7 Supplementary Figure 6: MSE between the algorithm results and the median scores of clinical pathologists for TPS in logarithmic scale.

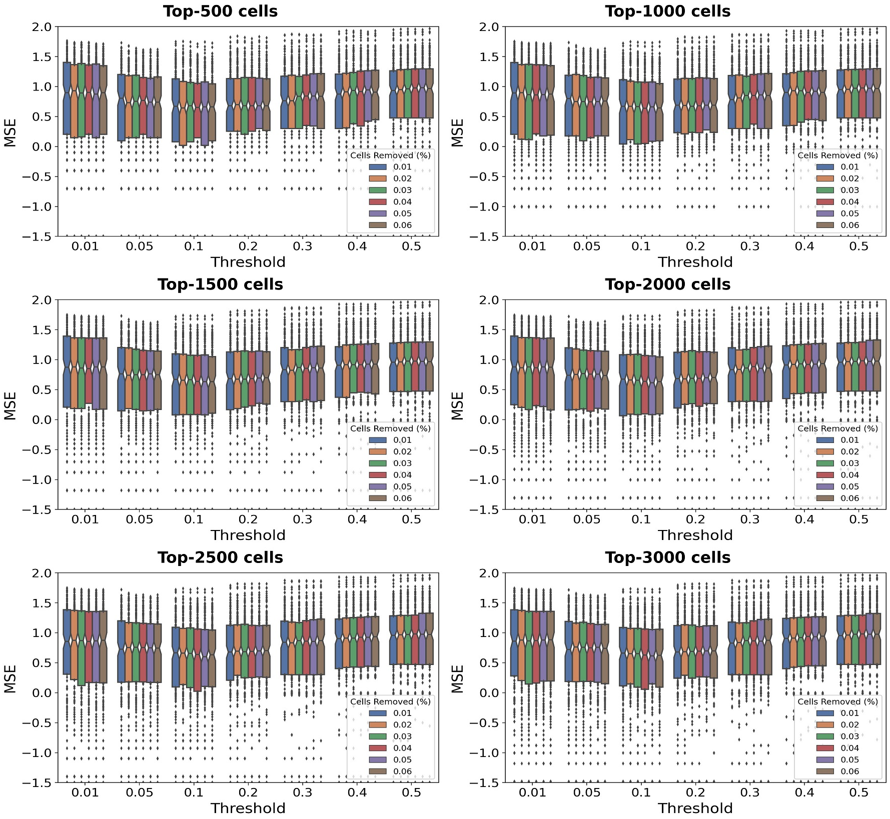

Fig. 6 The plot compares the impact of different parameter combinations on TPS. The parameters include the exclusion of the largest cells based on area percentage ($m\%$), selection of top cells with the largest area (k), and proportion of brown area in a single cell area ($t\%$). The 6 boxplots represent differences when k is set at 500, 1000, 1500, 2000, 2500, or 3000. Each boxplot contains 7 sets of comparisons with t values of 0.01, 0.05, 0.10, 0.20, 0.30, 0.40, or 0.50. Each set includes 6 results when m takes values of 0.01, 0.02, 0.03, 0.04, 0.05, or 0.06.

2.1.8 Supplementary Figure 7: Correlations between algorithm or pathologist results and mean, median scores of clinical pathologists.

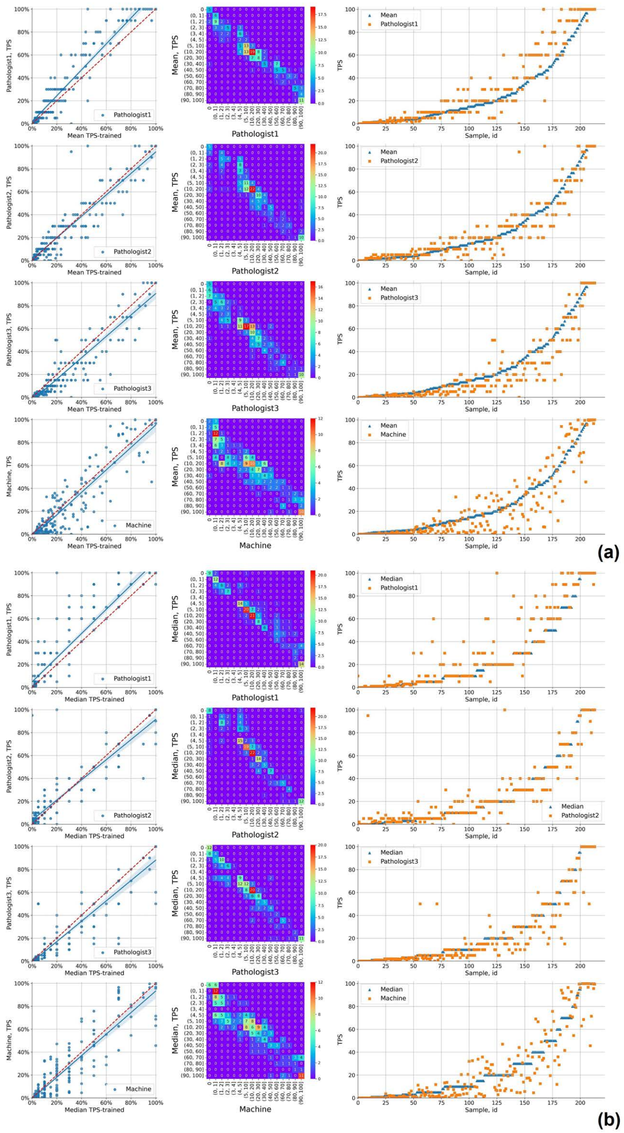

Fig. 7 Four rows represent the correlations between the TPS of clinical pathologists 1, 2, 3 and the algorithm with the mean (a) and median (b) scores of the three pathologists. Each row has three columns consisting of a scatter plot representing the correlation between individual and mean or median pathologist scores, a confusion matrix displaying the number of patients in each TPS interval, and a scatter plot of TPS for each sample from 220 individuals.

2.1.9 Supplementary Figure 8: Visualizations of correlations between algorithm or pathologist results and mean, median scores of clinical pathologists.

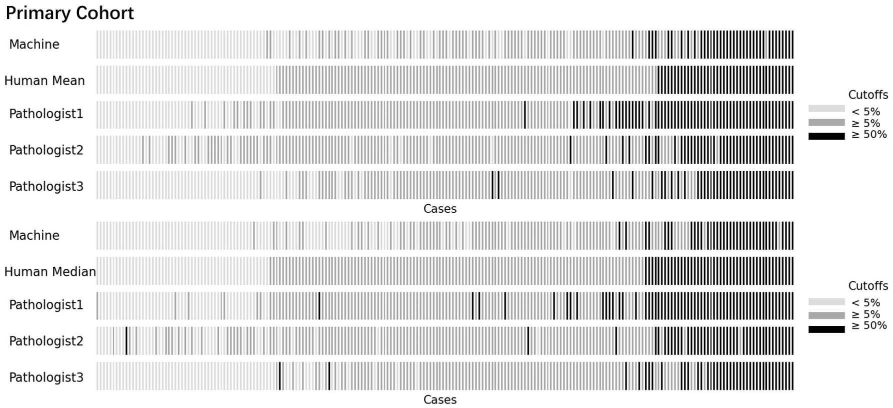

Fig. 8 TPS values are grouped into three intervals: less than 5% (represented by light gray), 5%-50% (represented by gray), and more than 50% (represented by dark gray). The correlations between TPS for clinical pathologists 1, 2, and 3, as well as the algorithm, and the mean (up), median (down) scores of the 3 pathologists are shown in 5 rows. Each row consists of 220 bars, with each bar representing a TPS interval.

2.2 Validation Cohorts (PD-L1)

2.2.1 Supplementary Figure 9: Visualizations of ROI segmentation in PD-L1 images.

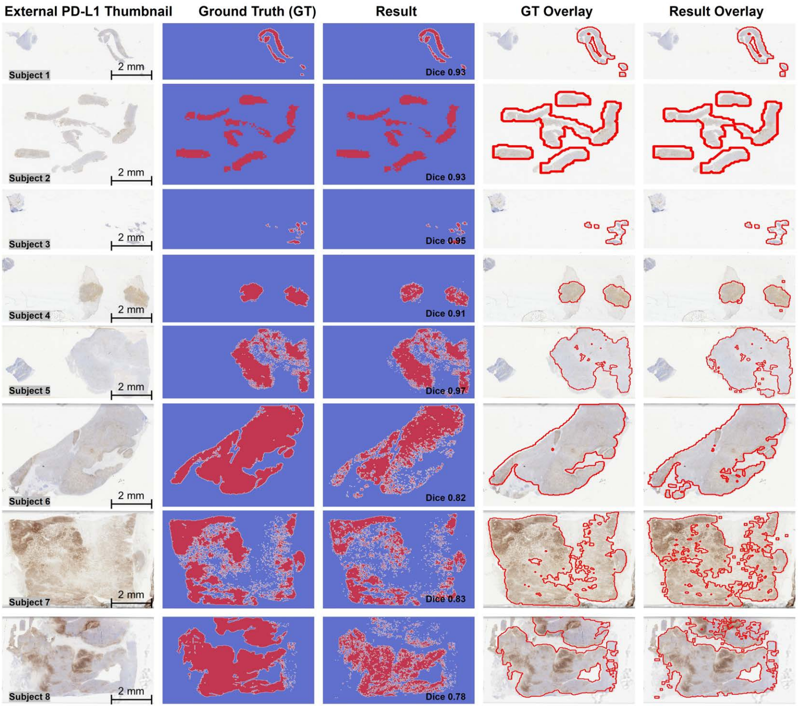

Fig. 9 This figure shows 8 sets of WSI-level results, selected from a dataset of 220 cases, in rows, with each row containing samples in the form of surgical specimens or fine needle biopsies. The figure is composed of five columns, which respectively display the thumbnail of a WSI, the binary heatmap of the GT annotated by experts, the binary heatmap of the algorithm results, the overlay of GT and the thumbnail, and the overlay of algorithm results and the thumbnail.

2.2.2 Supplementary Figure 10: Visualizations of cell center-point detection in PD-L1 Images.

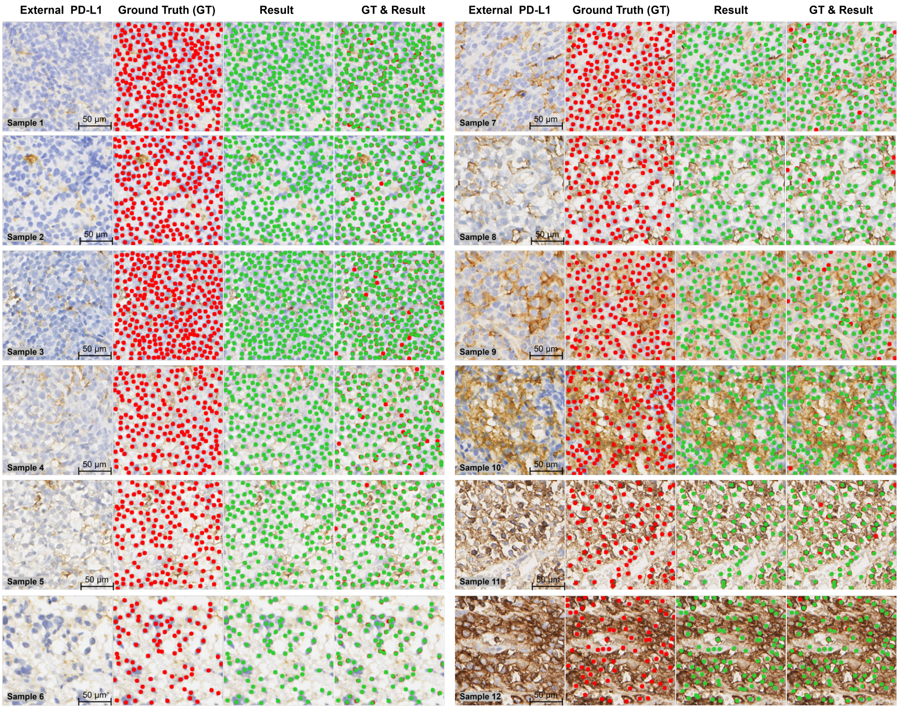

Fig. 10 A total of 12 groups of patch-level results, ranging from negative to strongly positive PD-L1 expression, were selected from the test set to display. Each set of results includes four columns, which are the original image, GT annotated by individuals with medical training, algorithm results, and the overlay of GT and algorithm results. Red and green dots are used to indicate the locations of cell centers detected by GT and algorithm results, respectively.

2.2.3 Supplementary Figure 11: Visualizations of cell segmentation in PD-L1 images.

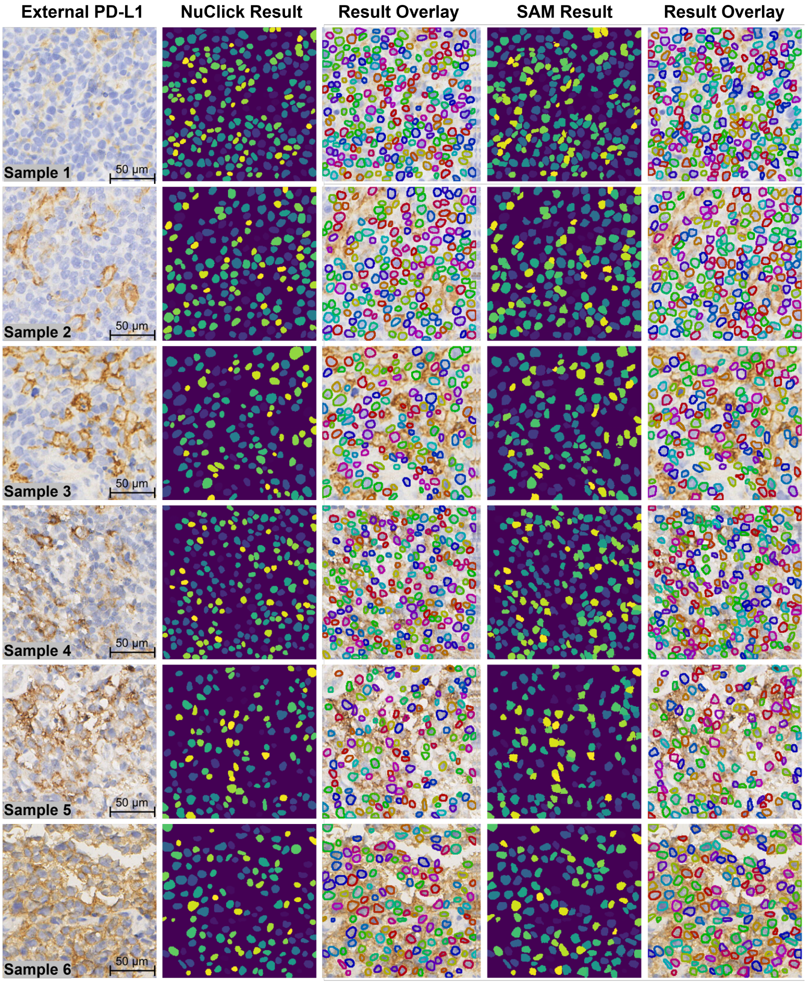

Fig. 11 This figure displays 12 groups of patch-level results, representing negative, weak positive, and strongly positive PD-L1 expression, selected from the test set. Each group of results includes three columns: the original image, the algorithm's segmentation results, and the overlay of the segmentation results and the original image. The different colors of the cell masks and contours are used solely for visualization purposes in the cell instance segmentation.

2.2.4 Supplementary Figure 12: Visualizations of negative and positive cell classification under various parameter thresholds.

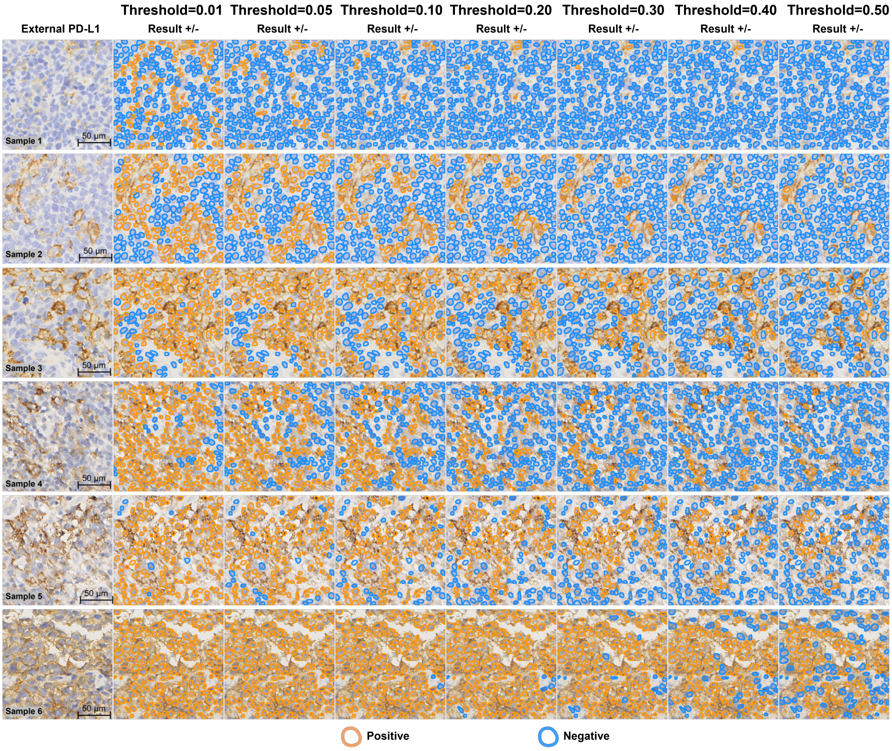

Fig. 12 Six groups of patch-level results, ranging from negative, weak positive, to strongly positive PD-L1 expression, were selected from the test set and displayed in each row. Each set of results consists of 8 columns, which are the original image and algorithm results with various parameters (i.e., 0.01, 0.05, 0.10, 0.20, 0.30, 0.40, 0.50) representing the proportion of brown area in a single cell region. The yellow circle in each patch represents the algorithm's determination that the PD-L1 expression of the cell is positive, while the blue circle represents that the expression is negative.

2.2.5 Supplementary Figure 13: Correlations between algorithm or pathologist results and mean, median scores of clinical pathologists.

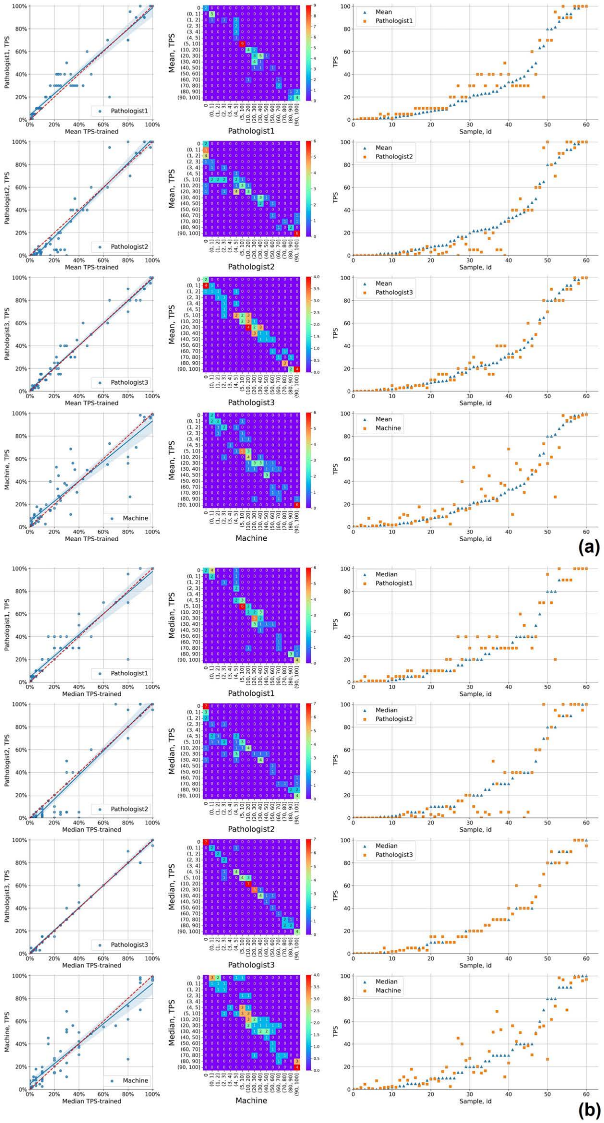

Fig. 13 Four rows represent the correlations between the TPS of clinical pathologists 1, 2, 3 and the algorithm with the mean (a) and median (b) scores of the three pathologists. Each row has three columns consisting of a scatter plot representing the correlation between individual and mean or median pathologist scores, a confusion matrix displaying the number of patients in each TPS interval, and a scatter plot of TPS for each sample from 61 individuals.

2.2.6 Supplementary Figure 14: Visualizations of correlations between algorithm or pathologist results and mean, median scores of clinical pathologists.

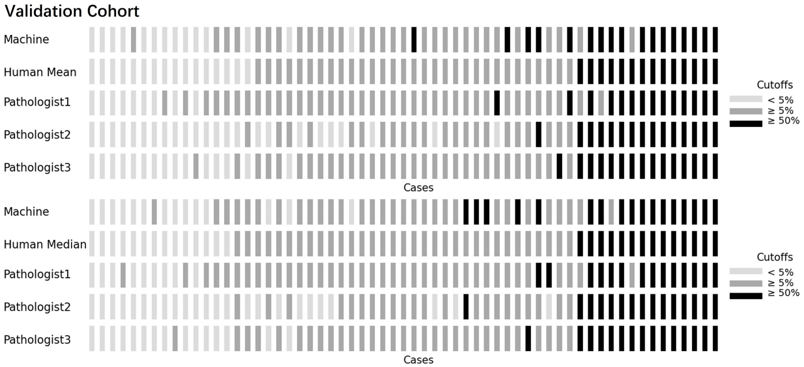

Fig. 14 TPS values are grouped into three intervals: less than 5% (represented by light gray), 5%-50% (represented by gray), and more than 50% (represented by dark gray). The correlations between TPS for clinical pathologists 1, 2, and 3, as well as the algorithm, and the mean (up), median (down) scores of the 3 pathologists are shown in 5 rows. Each row consists of 61 bars, with each bar representing a TPS interval.

2.2.7 Supplementary Figure 15: The interclass correlation coefficients (ICC) for PD-L1 expression between fine needle biopsies and surgical specimens

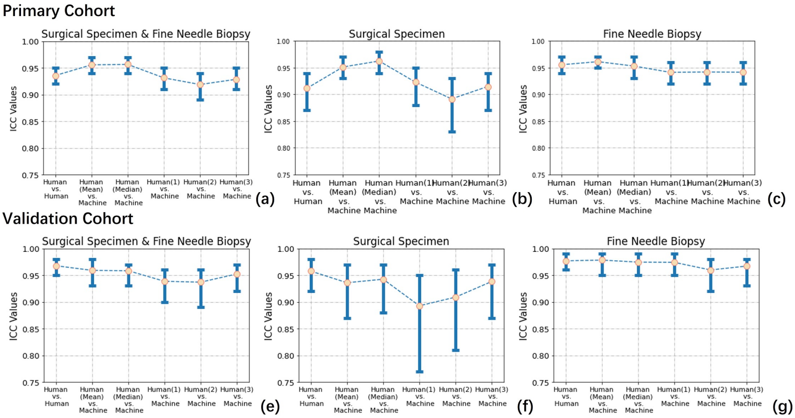

Fig. 15 The Interclass Correlation Coefficients (ICC) for PD-L1 Expression between Fine Needle Biopsies and Surgical Specimens. (a) shows the ICC values for both types of specimens, while (b) and (c) display the ICC values separately for surgical specimens and fine needle biopsies, respectively.

3 Methodology Supplement

3.1 Detailed Summary

WSIs display a pyramidal structure characterized by ultra-high resolution, posing significant challenges for algorithmic processing. We divided the foreground areas of WSIs into non-overlapping patches. Therefore, both our algorithmic analysis (including ROI segmentation, cell detection, cell segmentation and PD-L1 scoring) and data statistics were conducted based on these patches. For cell detection and segmentation, the performance of our algorithms can be assessed for each cell within these patches. However, in ROI Segmentation, it is imperative to correlate these patches back to the original WSIs using spatial coordinates to accurately evaluate algorithmic performance on entire WSIs. And the quantitative analysis of PD-L1 also necessitates the computation of the TPS directly from the complete WSIs.

Specifically, in ROI segmentation, we segment the foreground area of each WSI into patches, forming a bag with non-overlapping patches. The 220 WSIs from 220 patients were subdivided, resulting in approximately 1,057,486 patches in total. We conducted our algorithmic analysis in all WSIs including primary and validation cohorts. Compared to surgical specimens, fine needle biopsies contain a higher proportion of areas of interest for PD-L1 quantification, which is advantageous for the quantitative analysis of PD-L1. So we can acquire the ROI bag with patches from areas of interest and the non-ROI bag with patches from Non-ROIs for PD-L1 quantification. Only patches from the ROI bag are utilized in the quantitative analysis of PD-L1 for algorithms (as shown in Figure 16).

In cell detection, we solely utilize the ROI bag, which consists of each patch algorithmically determined to be within the ROIs, totaling approximately 674,361 patches. Since cell detection requires a model trained on annotated data, we selected 498 patches from this set, containing about 62,558 cells, for manual annotation. In PD-L1 scoring, three pathologists independently conducted quantitative evaluations on each slide from 220 PD-L1 slides in the primary cohort, resulting in a total of 660 (220*3) TPS (as shown in Tables 1 and 2).

Then we first segment out the cell nuclei and apply dilation to the segmentation mask of nuclei. The aim is to expand their coverage area to exceed the size of an individual membrane. The advantage of this approach lies in its ability to more accurately conform to the actual shape of the cell, thereby enhancing the accuracy of positive cell identification. During this process, we calculate the ratio of the brown area within the dilated cellular membrane boundary to the total area within this boundary. In the dilation process, the dilation kernel used is a 9x9 matrix of ones, and the number of iterations is 1 (as shown in Figure 17).

3.2 Supplementary Figure 16: Detailed description for multi-stage PD-L1 quantization.

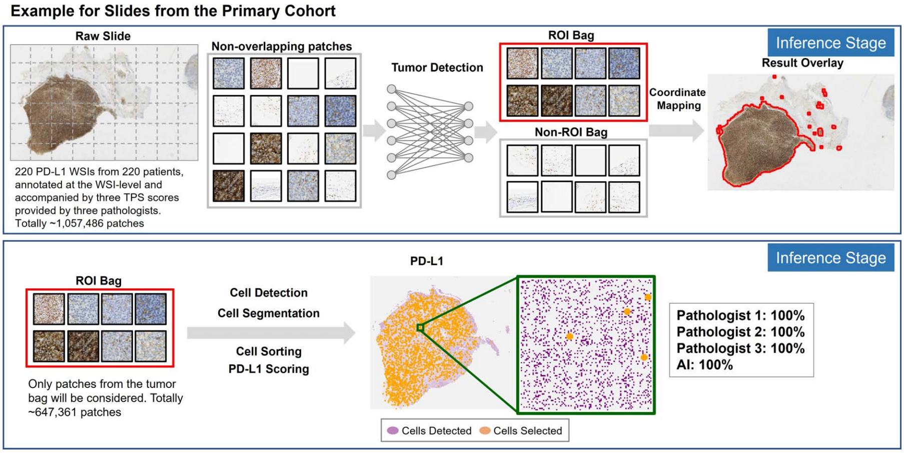

Fig. 16 Detailed description for multi-stage PD-L1 quantization.

3.3 Supplementary Figure 17: Dilation example in identifying positive cells.

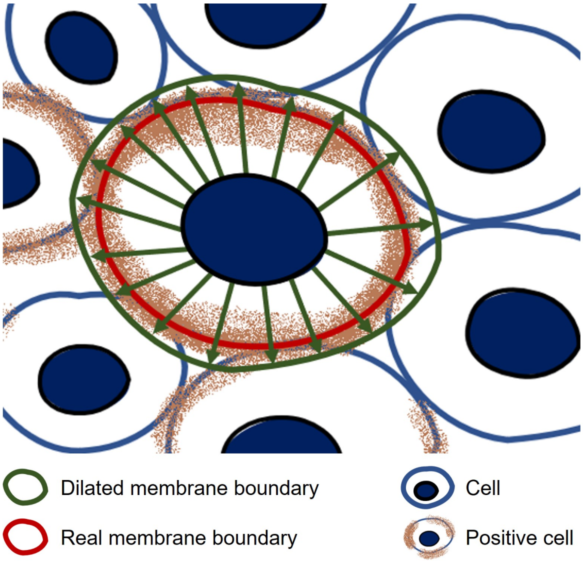

Fig. 17 Dilation example in identifying positive cells.

4 CD3 and CD10

Our two-stage cell instance segmentation framework was not limited to PD-L1 and can also be used for other IHC markers that involve membrane-positive IHC slides. This analysis incorporates diverse datasets including whole-slide images stained with CD3 and CD10. To ensure the coherence of the main text's logic, we included the content related to CD3 and CD10 in the supplementary material.

4.1 Data Profile

4.1.1 Supplementary Table 5: Data statistics of CD3

**Task**	**Level**	**Annotations**		**5-fold Cross-validation**			**Patients**
**Cell Detection**	Patch	471 patches	~47,749 cells	Infer	12 WSIs	471 patches	12
**Cell Segmentation**	Patch	-		Infer	12 WSIs	471 patches	12

Table 5 Data Statistics of CD3.

4.1.2 Supplementary Table 6: Data statistics of CD10

**Task**	**Level**	**Annotations**		**5-fold Cross-validation**			**Patients**
**Cell Detection**	Patch	504 patches	~87,740 cells	Infer	13 WSIs	504 patches	13
**Cell Segmentation**	Patch	-		Infer	13 WSIs	504 patches	13

Table 6 Data Statistics of CD10.

4.1.3 Annotations

CD3 (polyclone, DAKO) and CD10 (56C6, DAKO) were performed in a subset of 13 and 12 patients for cell detection and segmentation.

Two individuals (F.Y and M.F) with medical training labeled cell center points for CD3 and CD10 by LabelMe software [5] for the iteration of the cell detection algorithm. The selection method for CD3 and CD10 is the same as those for PD-L1 analysis. Finally, we obtained a total of 471 and 504 patches used for annotations of cell center points for CD3 and CD10, respectively.

4.2 Result Supplement

4.2.1 Supplementary Figure 18: Visualizations of cell center-point detection (CD3)

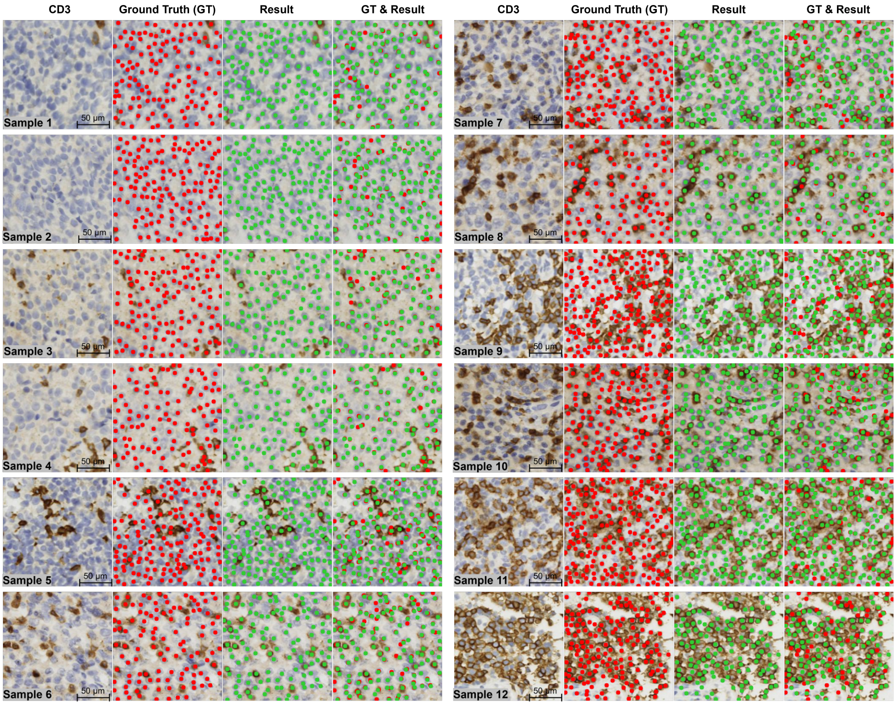

Fig. 18 Twelve groups of patch-level results covering negative, weak positive to strongly positive CD3 expression, are selected from the test dataset to display. Each set of results consists of 4 columns, which are the original image, GT annotated by individuals with medical training, algorithm results, and the overlay of GT and algorithm results. The red and green dots indicate the locations of the cell centers detected by GT and algorithm results, respectively.

4.2.2 Supplementary Figure 19: Visualizations of cell segmentation (CD3)

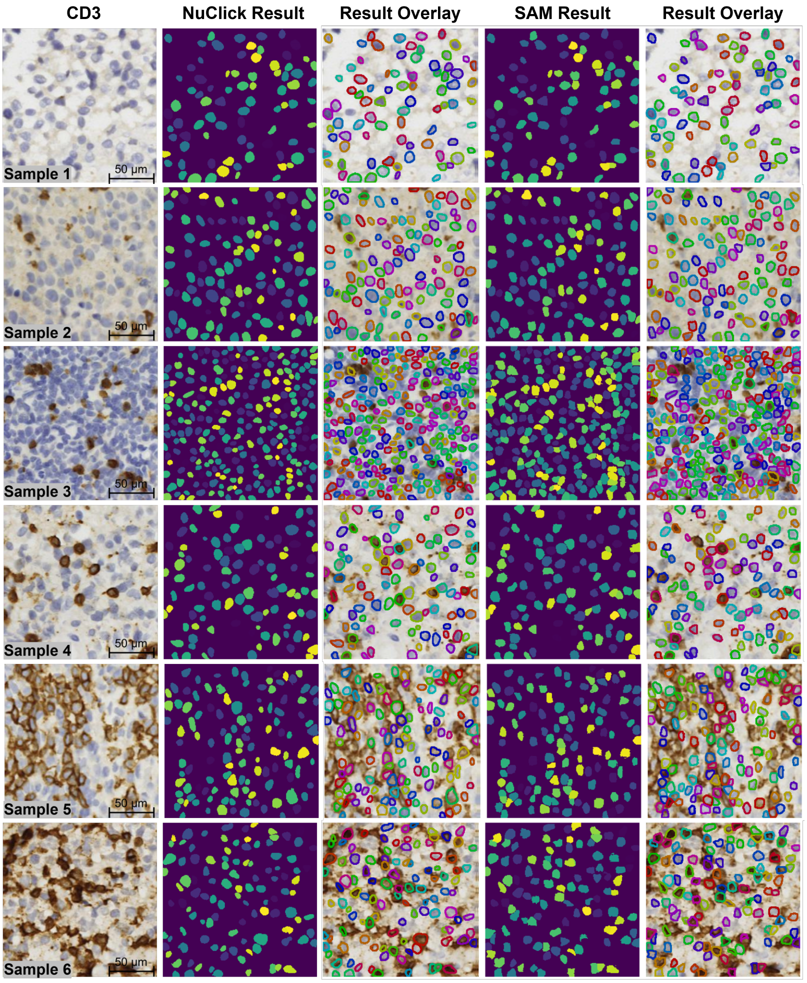

Fig. 19 Twelve groups of patch-level results covering negative, weak positive to strongly positive CD3 expression, are selected from the test dataset to display. Each set of results consists of 3 columns, which are the original image, algorithm results, and the overlay of algorithm results and the original image. The different colors of cell masks and contours are only used for visualization of cell instance segmentation.

4.2.3 Supplementary Figure 20: Visualizations of negative and positive cell classification under different parameter thresholds (CD3)

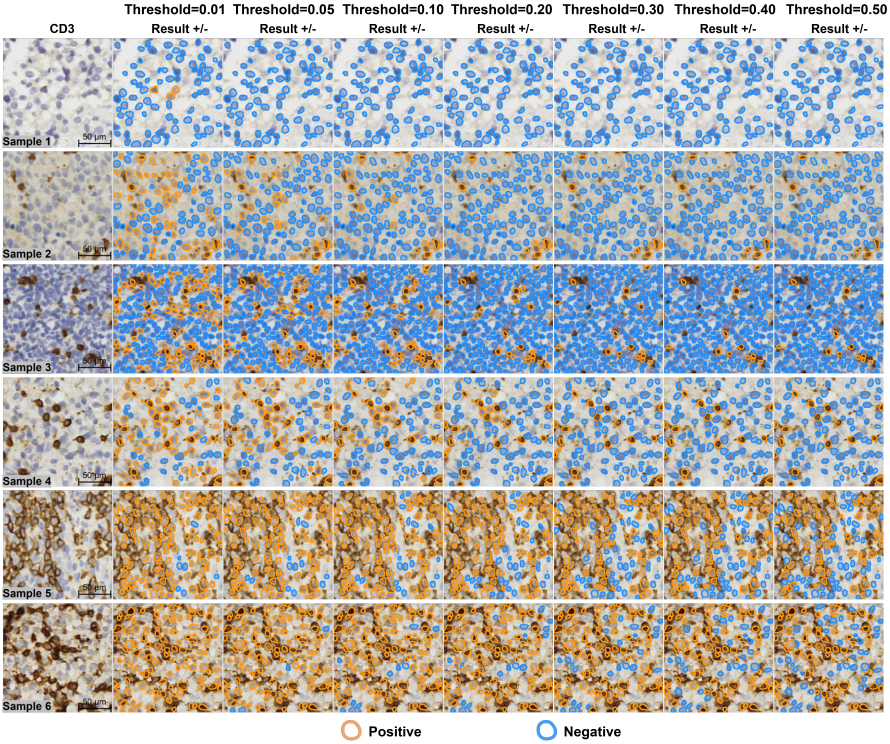

Fig. 20 Six groups of patch-level results covering negative, weak positive to strongly positive CD3 expression, are selected from the test set to display in each row. Each set of results consists of 8 columns, which are the original image, and algorithm results with the various parameters (i.e., 0.01, 0.05, 0.10, 0.20, 0.30, 0.40, 0.50) representing the proportion of brown area in a single cell region. The yellow circle in each patch represent that the algorithm determines that the CD3 expression of the cell is positive, while the blue circle represented that the expression is negative.

4.2.4 Supplementary Figure 21: Visualizations of cell center-point detection (CD10)

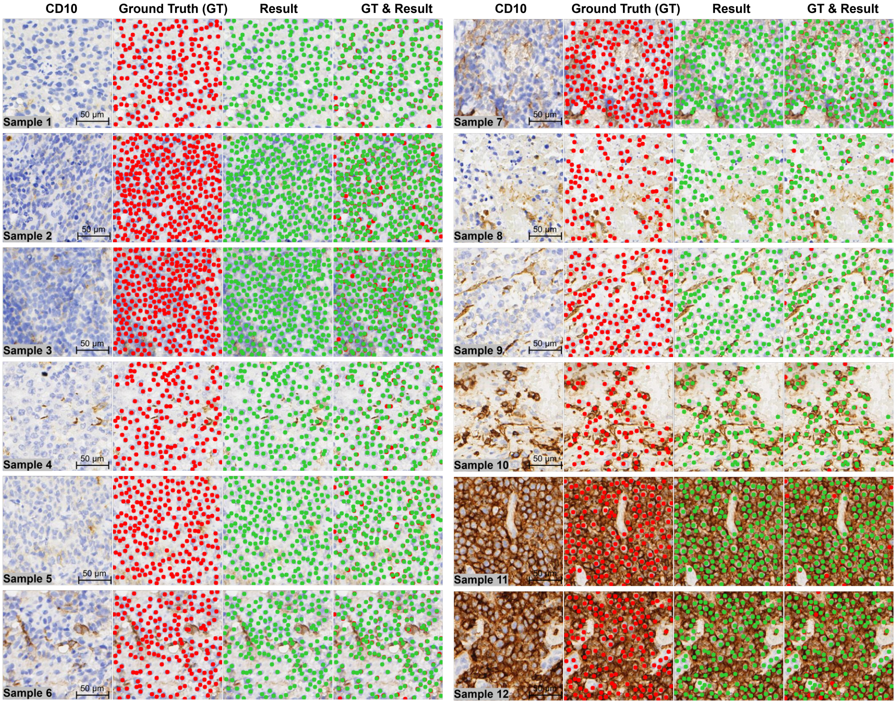

Fig. 21 Twelve groups of patch-level results covering negative, weak positive to strongly positive CD10 expression, are selected from the test dataset to display. Each set of results consists of 4 columns, which are the original image, GT annotated by individuals with medical training, algorithm results, and the overlay of GT and algorithm results. The red and green dots indicate the locations of the cell centers detected by GT and algorithm results, respectively.

4.2.5 Supplementary Figure 22: Visualizations of cell segmentation (CD10)

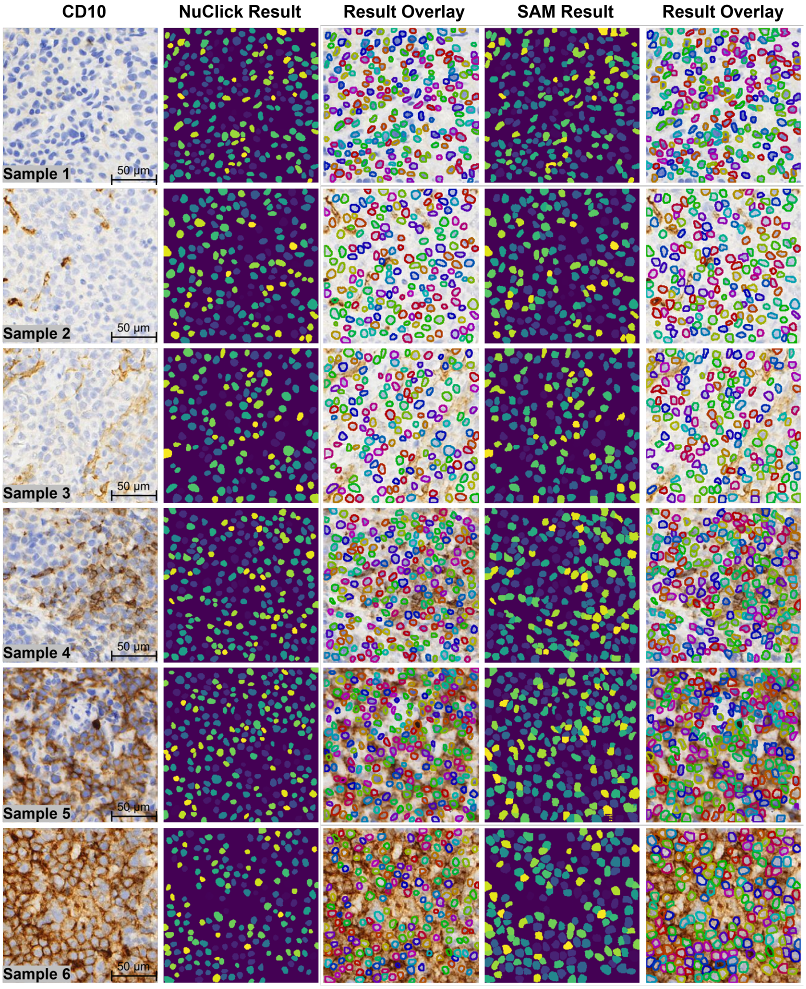

Fig. 22 Twelve groups of patch-level results covering negative, weak positive to strongly positive CD10 expression, are selected from the test dataset to display. Each set of results consists of 3 columns, which are the original image, algorithm results, and the overlay of algorithm results and the original image. The different colors of cell masks and contours are only used for visualization of cell instance segmentation.

4.2.6 Supplementary Figure 23: Visualizations of negative and positive cell classification under different parameter thresholds (CD10)

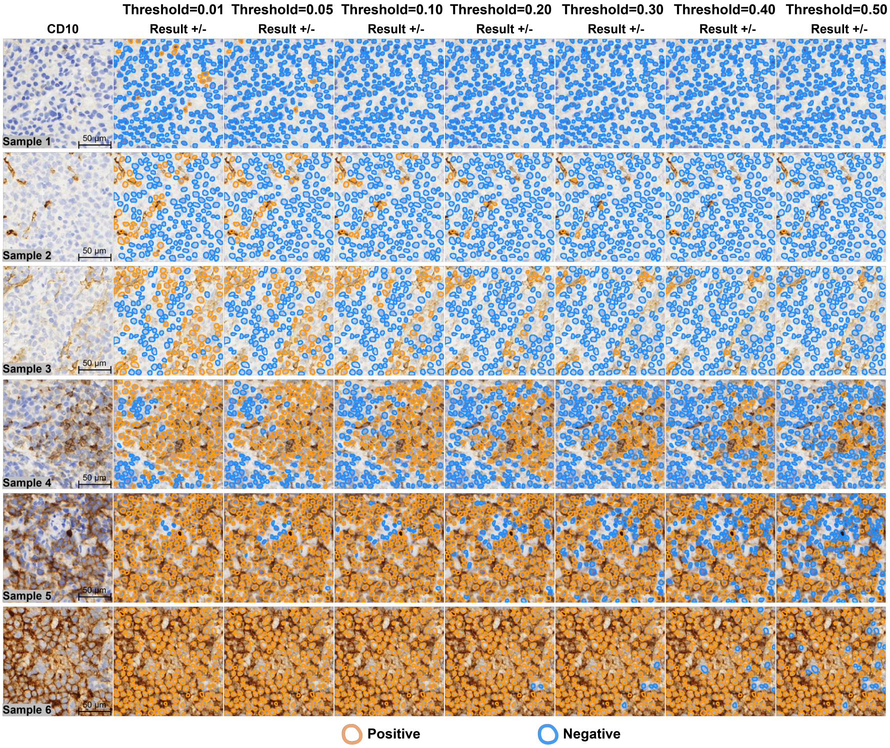

Fig. 23 Six groups of patch-level results covering negative, weak positive to strongly positive CD10 expression, are selected from the test set to display in each row. Each set of results consists of 8 columns, which are the original image, and algorithm results with the various parameters (i.e., 0.01, 0.05, 0.10, 0.20, 0.30, 0.40, 0.50) representing the proportion of brown area in a single cell region. The yellow circle in each patch represent that the algorithm determines that the CD10 expression of the cell is positive, while the blue circle represented that the expression is negative.

References

[1] Zhu, Y., Newsam, S.: Densenet for dense flow. 2017 IEEE International Conference on Image Processing (ICIP), 790–794 (2017)

[2] He, K., Zhang, X., Ren, S., Sun, J.: Deep residual learning for image recognition. Proceedings of the IEEE Conference on Computer Bision and Pattern Recognition, 770–778 (2016)

[3] Dosovitskiy, A., Beyer, L., Kolesnikov, A., Weissenborn, D., Zhai, X., Unterthiner, T., Dehghani, M., Minderer, M., Heigold, G., Gelly, S., Uszkoreit, J., Houlsby, N.: An image is worth 16x16 words: Transformers for image recognition at scale. The International Conference on Learning Representations (ICLR) (2021)

[4] Kirillov, A., Mintun, E., Ravi, N., Mao, H., Rolland, C., Gustafson, L., Xiao, T., Whitehead, S., Berg, A.C., Lo, W.-Y., Dollar, P., Girshick, R.: Segment anything. Proceedings of the IEEE International Conference on Computer Vision (ICCV), 4015–4026 (2023)

[5] Russell, B.C., Torralba, A., Murphy, K.P., Freeman, W.T.: Labelme: a database and web-based tool for image annotation. International Journal of Computer Vision **77**(1), 157–173 (2008)
